# Nanoscale metal-organic frameworks enhance radiotherapy to potentiate checkpoint blockade immunotherapy

**DOI:** 10.1038/s41467-018-04703-w

**Published:** 2018-06-15

**Authors:** Kaiyuan Ni, Guangxu Lan, Christina Chan, Bryan Quigley, Kuangda Lu, Theint Aung, Nining Guo, Patrick La Riviere, Ralph R. Weichselbaum, Wenbin Lin

**Affiliations:** 1 Department of Chemistry, The University of Chicago, Chicago, IL 60637 USA; 2 Department of Radiology, The University of Chicago, Chicago, IL 60637 USA; 3 Department of Radiation and Cellular Oncology and The Ludwig Center for Metastasis Research, The University of Chicago, Chicago, IL 60637 USA

## Abstract

Checkpoint blockade immunotherapy enhances systemic antitumor immune response by targeting T cell inhibitory pathways; however, inadequate T cell infiltration has limited its anticancer efficacy. Radiotherapy (RT) has local immunomodulatory effects that can alter the microenvironment of irradiated tumors to synergize with immune checkpoint blockade. However, even with high doses of radiation, RT has rarely elicited systemic immune responses. Herein, we report the design of two porous Hf-based nanoscale metal-organic frameworks (nMOFs) as highly effective radioenhancers that significantly outperform HfO_2_, a clinically investigated radioenhancer in vitro and in vivo. Importantly, the combination of nMOF-mediated low-dose RT with an anti-programmed death-ligand 1 antibody effectively extends the local therapeutic effects of RT to distant tumors via abscopal effects. Our work establishes the feasibility of combining nMOF-mediated RT with immune checkpoint blockade to elicit systemic antitumor immunity in non-T cell-inflamed tumor phenotypes without normal tissue toxicity, promising to broaden the application of checkpoint blockade immunotherapy.

## Abstract (web summary)

Radiotherapy is known to have local immunomodulatory effects, while an effective systemic immune response is an issue. Here, the authors report on nMOF radiosensitizers for enhanced radiotherapy which in combination with anti-PD-L1 antibodies result in a systemic immune response to distant tumors.

## Introduction

Cancer immunotherapy is becoming an important treatment modality alongside surgery, radiotherapy (RT), and chemotherapy for certain cancers.^1,2^ In its host-protective role, the immune system functions to detect and eliminate foreign entities, such as tumors. However, growing tumor masses can dysregulate signaling pathways, immune suppressive cells/cytokines, and effector molecules, thus preventing immune cells from recognizing and killing tumor cells.^3,4^ In checkpoint blockade immunotherapy, immunosuppressive pathways regulating T cells are blocked to enhance systemic antitumor immune responses.^5^ Programmed cell death protein 1 (PD-1) and its two ligands (PD-L1 and PD-L2) represent key pathways for immunosuppression.^6^ The interaction of PD-1 with either of its ligands inhibits kinase signaling pathways that are responsible for T cell activation, reducing effector T cell activity in tumors. Several anti-PD-1 and anti-PD-L1 antibodies have recently found clinical success in a subset of immunogenic tumors such as melanomas, non-small-cell lung cancer, and genitourinary cancers.^7–9^ However, targeting the PD-1/PD-L1 axis alone is insufficient to sustain an effective and durable response for most tumors, partly due to inadequate T cell infiltration into the cancerous tissues in non-immunogenic tumors.^10,11^ Therefore, immunomodulatory adjuvant treatments are actively pursued to synergize with checkpoint blockade immunotherapy to break immune tolerance and potentiate antitumor immunity in the host system.^12–14^

RT is a local treatment prevalently used across many cancer types in the clinic. High-dose, hypofractionated RT is studied as immunomodulatory adjuvant treatment to enhance checkpoint blockade immunotherapy in clinical trials.^15–18^ RT inflicts ionization damage to tumor tissues in an X-ray dose-dependent manner and its efficacy is usually limited by the maximum radiation dose that can be given to a tumor mass without incurring significant injuries to the neighboring tissues or organs.^19^ Conformation and/or intensity-modulated radiotherapies have been developed over the past few decades to provide greater spatial control on X-ray energy deposition, thus alleviating normal tissue toxicity.^20^ Reducing X-ray doses while maintaining sufficient ionization damage to tumors by using tumor-targeted radioenhancers can further minimize side effects to the surrounding tissues and also make RT a more compatible and effective adjuvant treatment to enhance checkpoint blockade immunotherapy.^21,22^

Heavy metal-based nanoparticles (NPs) such as Au and HfO_2_ NPs have been shown as promising radioenhancers.^23–26^ NPs of high atomic (*Z*) number elements have high X-ray absorption coefficients and, when selectively deposited in tumors, can significantly increase radiosensitivity difference between healthy and tumor tissues, thus reducing radiation dose without impacting anticancer activity. NP radioenhancers can thus potentially increase the therapeutic index of RT by sparing healthy tissues of high-dose radiation. For example, HfO_2_ NPs have been tested as a radioenhancer in several clinical trials for the treatment of soft tissue sarcomas, head and neck cancer, and other tumors.^27–29^ Yet, radiosensitizers with stronger radioenhancing effects are still needed for locally sensitizing tumors and eliciting immunomodulatory effects to enhance checkpoint blockade immunotherapy.^30,31^

Nanoscale metal-organic frameworks (nMOFs) have emerged as an important class of nanomaterials for biomedical applications due to their high porosity, multifunctionality, and biocompatibility.^32–35^ The ordered and porous structures of nMOFs have been shown to avoid self-quenching of photosensitizers and facilitate the diffusion of reactive oxygen species (ROS), leading to improved efficacy in photodynamic therapy. We hypothesized that electron-dense Hf-oxo-based secondary building units (SBUs) in nMOFs can preferentially absorb X-rays over tissues to enhance radiosensitization, while porous structures of nMOFs can facilitate the diffusion of generated ROS to exert cytotoxic effects, allowing for the design of a new generation of radioenhancers with superior radiotherapeutic efficacy.

Herein, we report a treatment strategy that combines nMOF-mediated RT and checkpoint blockade immunotherapy to achieve systemic rejection of colorectal tumors in mouse models. Two Hf-based nMOFs, Hf_6_-DBA and Hf_12_-DBA (DBA = 2,5-di(*p*-benzoato)aniline), are designed as radioenhancers by taking advantage of the electron-dense Hf_6_O_4_(OH)_4_ and Hf_12_O_8_(OH)_14_ SBUs as X-ray absorbers to generate ROS and the open channels as well as small nMOF dimensions to facilitate ROS diffusion. Hydroxyl radical generation studies show that both nMOFs display higher radioenhancing efficiencies than clinically investigated HfO_2_ NPs, which is supported by clonogenic assays and in vivo antitumor efficacy studies. These comparisons also reveal the superior efficacy of Hf_12_-DBA over Hf_6_-DBA, likely a result of enhanced ROS production due to increased X-ray absorption and more facile ROS diffusion owing to thin nanoplate structures. We also demonstrate in vivo that nMOF-mediated low-dose RT given in conjunction with an anti-PD-L1 antibody not only eradicates local tumors but also rejectes/regresses distant tumors through systemic antitumor immunity on a syngeneic CT26 colorectal cancer model. Furthermore, we profile the immune responses to probe the underlying mechanisms to provide further insight into this high level of abscopal effects from a combination of nMOF-mediated RT and checkpoint blockade immunotherapy.

## Results

### Synthesis and characterization of Hf_6_-DBA and Hf_12_-DBA nMOFs

**Fig. 1:**
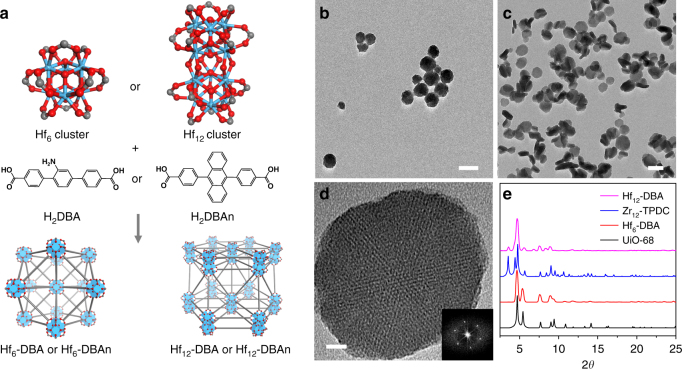
Structures and morphologies of Hf_6_-DBA and Hf_12_-DBA. **a** Synthesis of Hf_6_-DBA, Hf_6_-DBAn, Hf_12_-DBA, and Hf_12_-DBAn. Transmission electron microscopy (TEM) images of Hf_6_-DBA (**b**) and Hf_12_-DBA (**c**, **d**) with its fast Fourier transform (FFT) pattern shown in the inset of **d**. Scale bar = 100 nm (**b**, **c**) or 10 nm (**d**). **e** PXRD patterns of Hf_6_-DBA and Hf_12_-DBA nMOFs in comparison to those of UiO-68 and Zr_12_-TPDC. For TEM images, each nMOF was repeated at least five times and they all showed similar results

By tuning the temperature and modulators, two Hf-based nMOFs with different SBUs, Hf_6_-DBA with a formula of Hf_6_(*μ*_3_-O)_4_(*μ*_3_-OH)_4_(DBA)_6_ and Hf_12_-DBA with a formula of Hf_12_(*μ*_3_-O)_8_(*μ*_3_-OH)_8_(*μ*_2_-OH)_6_(DBA)_9_, were synthesized via solvothermal reactions (Fig. 1a).^35,36^ Transmission electron microscopy (TEM) imaging studies showed that Hf_6_-DBA exhibited spherical morphology with a diameter of approximately 68 nm, while Hf_12_-DBA possessed a plate-like morphology of ~98 nm in diameter and ~30 nm in thickness. Both nMOFs showed good dispersity in ethanol (Fig. 1b-d and Supplementary Figs. 1 and 2). Dynamic light scattering measurements gave a number-averaged diameter and polydispersity index of 116.0 ± 0.9 and 0.07 ± 0.01 nm, respectively, for Hf_6_-DBA and 102.1 ± 4.1 and 0.08 ± 0.02 nm, respectively, for Hf_12_-DBA (Supplementary Fig. 3). The powder X-ray diffraction (PXRD) pattern of Hf_6_-DBA was identical to that of UiO-68^36^, while the PXRD pattern of Hf_12_-DBA was identical to that of Zr_12_-TPDC^37^, indicative of their crystalline nature and different topological structures (Fig. 1e). Thermogravimetric analysis results of Hf_6_-DBA or Hf_12_-DBA matched the weight losses expected for their decomposition to HfO_2_ (Supplementary Fig. 4). Nitrogen sorption measurements showed that Hf_6_-DBA possessed a Brunauer–Emmett–Teller (BET) surface area of 804.4 m^2^/g, whereas Hf_12_-DBA exhibited a BET surface area of 463.9 m^2^/g (Supplementary Fig. 5). Ultra-small solid HfO_2_ NPs with a diameter of ~10 nm were also synthesized and used as a control (Supplementary Fig. 6).

Due to strong coordination between Hf^4+^ ions and carboxylate groups, both nMOFs were stable in aqueous suspensions. After incubation in RPMI-1640 cell culture medium for 240 h or upon X-ray irradiation at a dose of 16 Gy, the PXRD patterns of Hf_12_-DBA were identical to that of the pristine sample, indicating the structural stability of Hf_12_-DBA under physiological conditions and upon X-ray irradiation (Supplementary Figs. 7 and 8).

### Hydroxyl radical formation

**Fig. 2:**
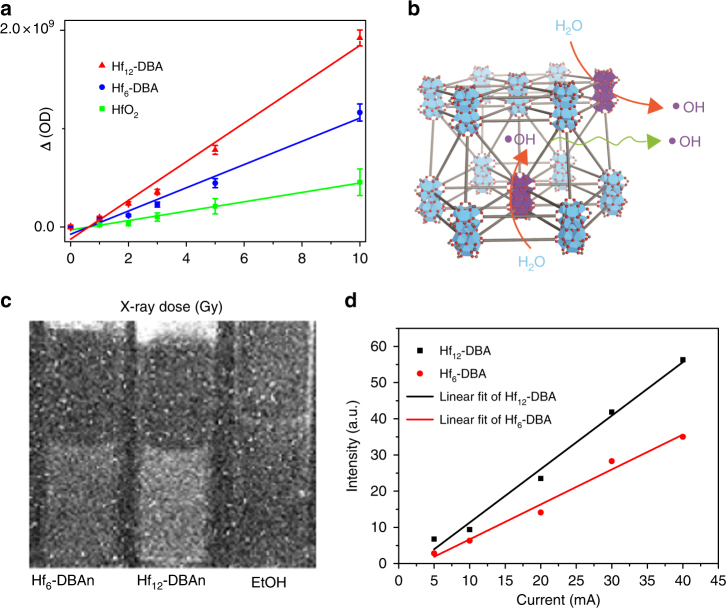
Radioenhancing efficiency of HfO_2_ vs. Hf_6_-based and Hf_12_-based nMOFs. **a** Enhanced APF fluorescence of HfO_2_, Hf_6_-DBA, and Hf_12_-DBA over H_2_O at equivalent Hf concentrations of 20 μM. *n* = 6. **b** Illustration of efficient hydroxyl radical generation upon X-ray irradiation and diffusion through porous Hf_12_-DBA nanoplates. **c** Optical images of radioluminescence from Hf_6_-DBAn (1 mM in ethanol), Hf_12_-DBAn (1 mM in ethanol), and pure ethanol at an X-ray dose rate of 2.93 Gy/min. **d** Linear fits of radioluminescence intensities with respect to X-ray tube currents of Hf_6_-DBA and Hf_12_-DBA after subtraction of pure ethanol background. Central data points and error bars represent mean ± s.d. values, respectively. Hydroxyl detection result was obtained without repetition. The radioluminescence images were obtained with two repetitions to afford similar results

Hydroxyl radicals, the major cytotoxic radical species from ionizing radiation, were detected via aminophenylfluorescein (APF) assay, in which APF reacts with hydroxyl radicals to give bright green fluorescence (excitation/emission maxima 490/515 nm). We first determined the percentage of APF trapped in Hf_6_-DBA or Hf_12_-DBA via detecting chemically produced hydroxyl radicals from Fenton’s reaction by APF with and without the nMOF: the percentage of APF trapped in Hf_6_-DBA or Hf_12_-DBA equals to one minus the fluorescence intensity ratio of APF with nMOF over that of APF without nMOF (Supplementary Method 2 and Supplementary Figs. 9 and 10). We then irradiated 5 μM APF in aqueous solution or in aqueous dispersion of HfO_2_, Hf_6_-DBA, or Hf_12_-DBA at an Hf concentration of 20 μM with X-ray in the dose range of 0 to 10 Gy and determined their fluorescence signals. After correcting for the percentage of APF trapped in the nMOF, we deduced the fluorescence intensity corresponding to the amounts of hydroxyl radicals generated by water, HfO_2_, Hf_6_-DBA, and Hf_12_-DBA as shown in Supplementary Fig. 10. All four groups showed linear increases of hydroxyl radical generation with increasing X-ray dose. The relative enhancement of hydroxyl radical generation compared with water was obtained by subtracting the APF fluorescence intensity from water as shown in Fig. 2a. Compared to water, HfO_2_, Hf_6_-DBA, and Hf_12_-DBA enhanced hydroxyl generation by 14.3%, 33.6%, or 55.3%, respectively.

### Radioluminescence

Upon X-ray irradiation, Hf-based MOFs were previously shown to transfer energy to anthracene-based bridging ligands, DBAn (DBAn = 2,5-di(*p*-benzoato)antheracene), to emit radioluminescence in the visible spectrum.^32^ Radioluminescence of Hf-DBAn-based MOFs can thus be used to probe their X-ray absorption efficiency. We synthesized Hf_6_-DBAn and Hf_12_-DBAn, two DBAn MOFs with Hf_6_ and Hf_12_ SBUs, for radioluminescence measurements to investigate the relationship between SBU structure and X-ray absorption efficiency (Supplementary Figs. 11 and 12). After degassing, 4 mL vials of Hf_12_-DBAn and Hf_6_-DBAn at an Hf concentration of 1 mM in ethanol along with the ethanol control were irradiated with X-rays at a maximum dose rate of 2.93 Gy/min and their radioluminescence images were acquired using a CCD camera. As shown in Fig. 2c, Hf_12_-DBAn gave a much brighter radioluminescence signal compared to Hf_6_-DBAn and EtOH. Image processing software (ImageJ) was then used to calculate radioluminescence intensities by sampling the average pixel value of the vials and subtracting the average pixel value of pure ethanol background. The measured intensities were fit linearly as a function of X-ray tube current, which is proportional to the radiation dose (Fig. 2d). Hf_12_-DBAn had radioluminescence slope of 1.36 ± 0.05, 1.58-fold higher than that of Hf_6_-DBAn (0.86 ± 0.04, Supplementary Table 1). This result indicated that Hf_12_-DBAn exhibited approximately 1.5 times higher X-ray absorption efficiency than Hf_6_-DBAn. Both hydroxyl radical generation and radioluminescence measurements thus demonstrated that Hf_12_-DBA is an excellent radioenhancer for RT.

### Clonogenic assay

Time-dependent cellular uptake of HfO_2_, Hf_6_-DBA, and Hf_12_-DBA in CT26 cells from 1 to 8 h demonstrated efficient cellular uptake of nMOFs and 4 h incubation was chosen as the time point for further in vitro studies (Supplementary Method 2 and Supplementary Fig. 13). Facile nMOF endocytosis was confirmed by confocal imaging; nMOFs labeled with Rhodamine B (Hf_6_-DBA-R and Hf_12_-DBA-R) co-localized with Lysotracker Green that labeled endo/lysosomes in both Hf_6_-DBA-treated or Hf_12_-DBA-treated cells (Supplementary Method 3 and Supplementary Fig. 14).

Clonogenic assays were performed to assess the colony-forming potential of cells treated with nMOFs at an Hf concentration of 20 µM for 4 h followed by irradiation with either X-ray or ^60^Co isotope γ-ray source at 0–16 Gy. Treated cells were trypsinized, re-seeded, and cultured for 10–20 days and the clones were counted and plotted with the survival fraction as shown in Supplementary Figs. 15 and 16. Radiation enhancement factor at 10% survival dose (REF_10_) was calculated as the ratio of equivalent irradiation doses needed to give 10% survival rate for the phosphate-buffered saline (PBS) control group over that for the experimental group.

As shown in Table 1, 20 μM HfO_2_ showed only moderate radioenhancing effect compared to PBS and exhibited much smaller REF_10_ values than Hf_6_-DBA and Hf_12_-DBA in all cell lines examined, which is consistent with the APF assay results. Interestingly, at the same Hf concentration, Hf_12_-DBA outperformed Hf_6_-DBA, with REF_10_ values from 1.45 to 1.73 compared to those from 1.10 to 1.31 for Hf_6_-DBA. Hf_12_-DBA thus exhibited superior radioenhancement over Hf_6_-DBA, likely due to enhanced X-ray absorption by the electron-dense Hf_12_ clusters and hydroxyl radical diffusion through the porous nanoplates (Fig. 2b). Upon irradiation with γ-rays from a ^60^Co source, Hf_12_-DBA also exhibited higher radiosensitization (REF_10_ = 1.10–1.47) than HfO_2_ and Hf_6_-DBA, suggesting that Hf_12_-DBA is compatible with linear accelerators commonly used in the clinic.

**Table 1:** REF_10_ values by clonogenic assays in a panel of cell lines upon X-ray irradiation or γ-ray irradiation from a ^60^Co source

	REF_10_	4T1	TUBO	HeLa	CT26	JSQ3	SQ20B
X-ray	HfO_2_	1.09	1.13	1.10	1.03	1.00	1.16
	Hf_6_-DBA	1.11	1.31	1.25	1.19	1.10	1.29
	Hf_12_-DBA	1.45	1.73	1.43	1.49	1.65	1.56
γ-ray	HfO_2_	1.06	1.03	1.11	1.01	1.00	1.00
	Hf_6_-DBA	1.15	1.27	1.31	1.06	1.06	1.08
	Hf_12_-DBA	1.26	1.44	1.47	1.14	1.10	1.23

### DNA double-strand break analysis

**Fig. 3:**
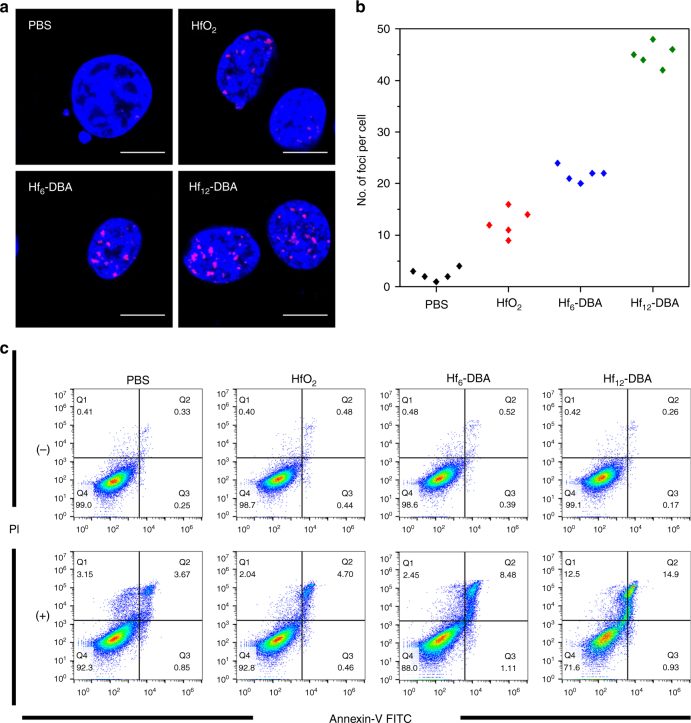
In vitro mechanistic studies of nMOF-mediated RT cytotoxicity. **a** γ-H2AX assays showing the DSBs in CT26 cells treated with nMOFs and X-ray irradiation. Blue and red fluorescences show DAPI-stained nucleus and antibody-labeled γ-H2AX in the cells, respectively. Scale bar = 10 µm. **b** Quantitative analysis of number of DBS foci per nucleus. **c** Annexin V/PI analysis of CT26 cells. Cells were incubated with PBS, HfO_2_, Hf_6_-DBA, or Hf_12_-DBA with or without X-ray irradiation at a dose of 4 Gy. The quadrants from lower left to upper left (counter clockwise) represent healthy, early apoptotic, late apoptotic, and necrotic cells, respectively. The percentage of cells in each quadrant was shown on the graphs. (+) and (−) refer to with and without irradiation, respectively. Both confocal images and flow cytometry results were obtained with two repetitions. One of two repetitions with similar results is shown

To elucidate the anticancer mechanism of nMOF-based RT, we investigated DNA double-strand break (DSB) and cell death pathways caused by nMOFs upon X-ray irradiation in CT26 cells. γ-H2AX, a phosphorylated protein that resulted from direct ionizing radiation or generated hydroxyl radicals to induce DNA damage repair, has been used as a sensitive biomarker for probing DSBs.^38,39^ Twenty-four hours after irradiation, significant red γ-H2AX fluorescence indicating DSBs was observed in the groups treated with Hf-based NPs, while no fluorescence was observed in groups either without X-ray irradiation or without Hf-based NP (Supplementary Fig. 17). Hf-based NP-treated groups also showed significant increases in the percentage of cells with DSB foci compared with the PBS control (Supplementary Fig. 18). Quantitative flow cytometric analyses showed that cells treated with Hf_12_-DBA exhibited stronger red fluorescence than cells treated with Hf_6_-DBA or HfO_2_, confirming that Hf_12_-DBA induced stronger DNA DSB (Supplementary Fig. 19). Hf_12_-DBA additionally induced more foci per cell as shown in Fig. 3a, with 45.0 ± 2.2, 21.8 ± 1.5, 12.4 ± 2.7, and 2.4 ± 1.1 foci per nucleus in Hf_12_-DBA-treated, Hf_6_-DBA-treated, HfO_2_-treated, and PBS treated cells, respectively (Fig. 3b). These results support potent radioenhancing efficiency of the unique Hf_12_ structure.

### Apoptotic cell death analysis

The cell death pathways were then evaluated with Annexin V/Dead Cell Apoptosis Kit. Significant amounts of cells underwent apoptosis/necrosis when treated with Hf_12_-DBA and X-ray irradiation with only 71.6% healthy cells, compared to 92.8% and 88.0% healthy cells for HfO_2_ or Hf_6_-DBA plus X-ray irradiation (Fig. 3c). 90%+ cells remained healthy in dark controls or the PBS group with irradiation, indicating that NP radioenhancers are not intrinsically cytotoxic and the low-dose X-ray showed negligible cytotoxicity without a NP radioenhancer. Taken together, Hf_12_-DBA is a significantly more efficient radioenhancer than both HfO_2_ and Hf_6_-DBA at equivalent Hf and X-ray doses.

### In vivo antitumor efficacy

After demonstrating that Balb/c mice dosed subcutaneously with 10 µmol Hf_12_-DBA (based on Hf) showed no difference in body weight evolution compared with PBS control (Supplementary Fig. 20), we carried out in vivo efficacy studies with mice receiving a single nMOF injection followed by X-ray irradiation. A colorectal adenocarcinoma mouse model of single CT26 tumor-bearing BALB/c mice was employed to evaluate the radioenhancing effects and antitumor efficacy of HfO_2_, Hf_6_-DBA, and Hf_12_-DBA. When the tumors reached 100–150 mm^3^ in volume, Hf_6_-DBA, Hf_12_-DBA, or HfO_2_ NPs were injected intratumorally at equivalent Hf doses of 1 µmol followed by daily X-ray irradiation at a dose of 1 Gy/fraction (120 kVp, 20 mA, 2 mm Cu filter) for a total of 10 fractions on consecutive days. An additional high-dose group of HfO_2_ at 3.2 µmol was employed to further illustrate the difference between nMOFs and HfO_2_.

**Fig. 4:**
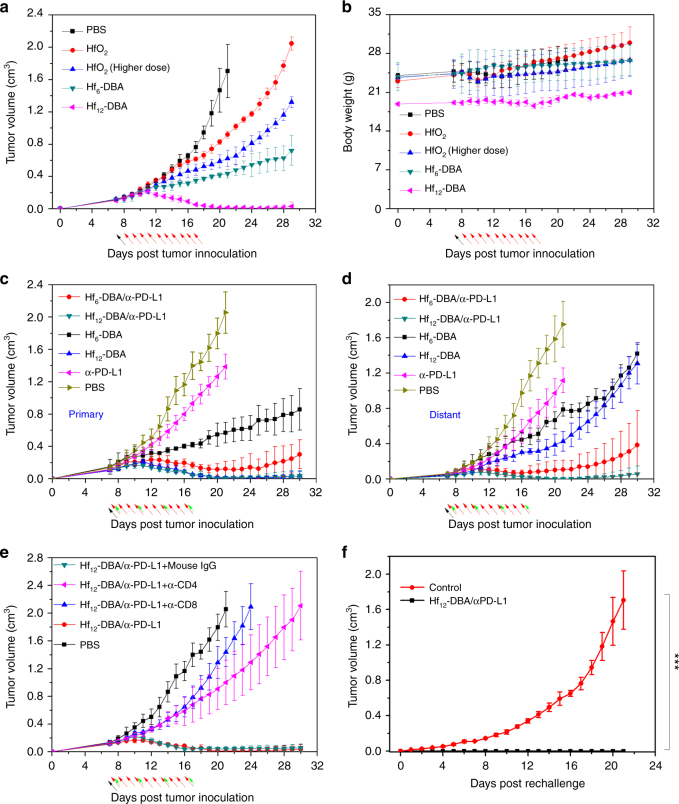
In vivo studies comparing antitumor efficacy of Hf-based NPs. Tumor growth inhibition curves (**a**) and body weights (**b**) after RT treatment in the single CT26 tumor-bearing mice treated with PBS, HfO_2_ (low and high doses), Hf_6_-DBA, or Hf_12_-DBA. Tumor growth curves of **c** primary tumors and **d** distant tumors of CT26 bilateral tumor-bearing mice treated with Hf_6_-DBA (with or without anti-PD-L1 antibody), Hf_12_-DBA (with or without anti-PD-L1 antibody), anti-PD-L1 antibody, or PBS with X-rays irradiation. **e** Tumor growth curves of CT26 tumor-bearing mice with T cell depletion and treated with Hf_12_-DBA, anti-PD-L1 antibody, and X-ray irradiation. **f** Tumor growth curves after the tumor rechallenge with CT26 cells. Treatment began on day 7 after tumor inoculation when the tumor reached a volume of 100–150 mm^3^. X-ray irradiation was carried out on mice 12 h after the i.t. injection of PBS or NPs on 10 consecutive days at a dose of 1 Gy/fraction (120 kVp, 20 mA, 2 mm Cu filter). Antibody was given intraperitoneally every 3 days at a dose of 75 µg/mouse. Black, red, and green arrows refer to the times of PBS or nanoparticles injections, X-ray irradiation, and antibody administration, respectively. *n* = 6. *P* values for comparisons with controls by *t* test are indicated by three asterisks: ***P < 0.001. Central data points and error bars represent mean ± s.d. values, respectively. The Hf_12_-DBA with anti-PD-L1 treatment group was repeated once

As shown in Fig. 4a, two Hf-based nMOFs afforded better local RT outcomes than HfO_2_. Hf_12_-DBA effectively regressed the locally irradiated tumors after a total of 10 Gy X-ray irradiation. In comparison, Hf_6_-DBA-treated and HfO_2_-treated groups showed only moderate anticancer efficacy, even after increasing the HfO_2_ dose 3.2-fold. The body weights of mice remained consistent regardless of treatment (Fig. 4b), suggesting that there was no systemic toxicity. Inductively coupled plasma-mass spectrometry analysis showed that the amounts of Hf_12_-DBA in tumors slowly decreased following intratumoral injection (Supplementary Fig. 21).

### In vitro and in vivo evaluation of immunogenicity

**Fig. 5:**
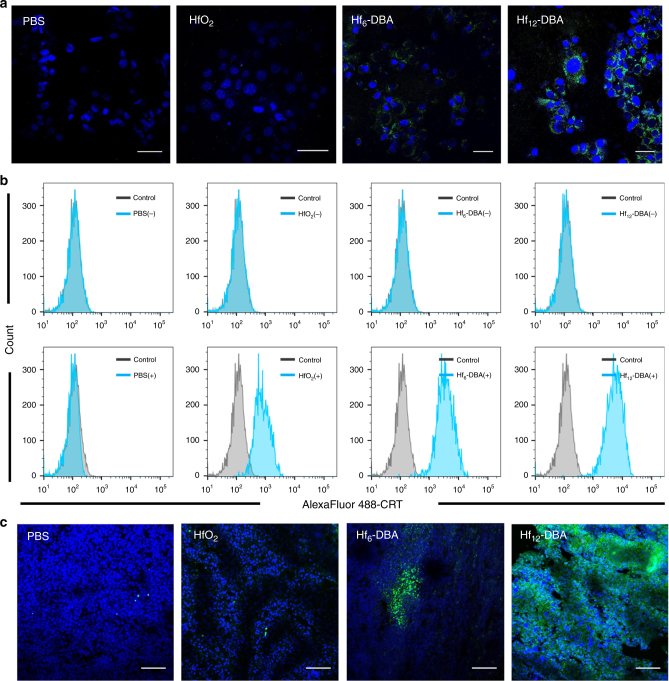
In vitro and in vivo immunogenic cell death studies. In vitro CRT exposure on the cell surface of CT26 was assessed after incubation with PBS, HfO_2_, Hf_6_-DBA, or Hf_12_-DBA with X-ray irradiation by immunofluorescence microscopy (**a**) and flow cytometry (**b**). (+) and (−) refer to with and without irradiation, respectively. **c** In vivo CRT exposure was evaluated on sectioned tumor slides of CT26 tumor-bearing mice after 5 consecutive days of X-ray irradiation. Both in vitro confocal images and flow cytometry analyses were obtained with two repetitions. One of two repetitions with similar results is shown. The in vivo confocal images were obtained without repetition. Scale bar = 20 µm (**a**) or 100 µm (**c**)

The immunogenic cell death (ICD) induced by Hf NP-mediated radiation treatment was investigated by detecting cell-surface expression of calreticulin (CRT) both in vitro and in vivo.^40^ As shown in Fig. 5a and Supplementary Fig. 22, more green fluorescence was observed in the group treated with nMOFs compared to groups treated with either PBS or HfO_2_ under confocal laser scanning microscopy (CLSM), suggesting higher immunogenicity of nMOF-mediated RT treatment. Quantitative flow cytometry analyses confirmed that Hf_12_-DBA showed significantly higher CRT expression on the cell-surface upon irradiation compared to Hf_6_-DBA, HfO_2_, PBS, or without irradiation (Fig. 5b), demonstrating that Hf_12_-DBA-mediated radiation treatment induced stronger ICD over groups treated with either Hf_6_-DBA or HfO_2_ (Supplementary Fig. 23).

We also evaluated CRT expression in CT26 tumor-bearing mice treated with PBS, HfO_2_, Hf_6_-DBA, or Hf_12_-DBA and 5 consecutive days of irradiation at 1 Gy/fraction. The tumors were excised and sectioned for immunostaining. Hf_12_-DBA-treated tumors showed more green fluorescence (Fig. 5c), indicating that the superior radioenhancement of Hf_12_-DBA also led to higher CRT expression in vivo.

To further demonstrate the ICD, HMGB1 excretion was examined by the enzyme-linked immunosorbent assay assay in vitro. Compared to groups treated with either Hf_6_-DBA or HfO_2_, the higher excretion of HMGB1 from cells treated with Hf_12_-DBA provided additional evidence for the notion that Hf_12_-DBA-mediated radiation treatment induced stronger ICD (Supplementary Fig. 24).

### In vivo antitumor efficacy of nMOF-mediated RT plus immune checkpoint blockade

We then combined nMOF-mediated RT with checkpoint blockade immunotherapy to extend local treatment to systemic cancer management. A bilateral model of CT26 was established to assess the systemic anticancer efficacy of Hf_6_-DBA-mediated or Hf_12_-DBA-mediated RT combined with immune checkpoint blockade. When the primary tumors reached 100–150 mm^3^ in volume, nMOFs were intratumorally injected to the primary tumors at equivalent doses of 1 µmol Hf, followed by daily X-ray irradiation at a dose of 1 Gy/fraction (120 kVp, 20 mA, 2 mm Cu filter) for a total of 10 fractions on consecutive days. Seventy-five micrograms of anti-PD-L1 antibody was given every 3 days by intraperitoneal injection.

Combination treatment of nMOFs and anti-PD-L1 antibody regressed the locally irradiated tumors at 10 Gy of total X-ray dose. Again, the stronger radioenhancer Hf_12_-DBA outperformed Hf_6_-DBA in combination therapy (Fig. 4c). More significantly, when combined with an anti-PD-L1 antibody, Hf_12_-DBA not only produced local regression of irradiated tumors but also shrank distant, non-irradiated tumors. The inhibition of distant tumors by Hf_12_-DBA-mediated RT plus checkpoint blockade therapy indicated effective induction of systemic antitumor immune response (Fig. 4d). Histological analysis of the tumors confirmed nMOF-mediated RT caused apoptosis/necrosis in local tumors, while only Hf_12_-DBA/anti-PD-L1 antibody-treated group showed apoptotic tumor histology with a lower density of tumor cells in the untreated distant tumor (Supplementary Fig. 25). The histologies of major organs did not significantly differ from those of PBS-treated mice, indicating that nMOF-mediated RT with or without checkpoint blockade did not lead to systemic toxicity (Supplementary Fig. 26). This was further confirmed by the steady body weights in all treated mice (Supplementary Fig. 27).

### Depletion studies

We further investigated how the therapeutic effects of nMOF-mediated RT plus anti-PD-L1 treatment were affected by immune cells. The anticancer efficacy of combination therapy was assessed in the subcutaneous CT26 model with concurrent depletion of CD4^+^ T or CD8^+^ T cells. Mice were intraperitoneally injected with anti-CD4, anti-CD8, or mouse IgG antibodies and then treated with Hf_12_-DBA and X-ray irradiation combined with an anti-PD-L1 antibody. Both anti-CD4 and anti-CD8 antibody-treated groups showed rapid tumor growth after cessation of X-ray treatment, but mouse IgG treatment did not have any effect on tumor growth (Fig. 4e). These results demonstrated that both CD4^+^ T and CD8^+^ T cells played an essential role in the anticancer efficacy of our combination treatment, supporting the rationale of using nMOF-mediated RT to enhance checkpoint blockade cancer immunotherapy.

### Tumor challenge studies

To confirm the long-term antitumor immune response, we carried out a tumor challenge study. CT26 tumors were established on the right flanks of mice and treated with Hf_12_-DBA and X-ray (1 Gy/fraction, 10 fractions in consecutive days). Three out of six mice had their tumors completely eradicated after treatment, affording a cure rate of 50%. Tumors in the other three mice shrank to very small sizes, but regrew beginning days 17, 26, and 32, respectively, post tumor inoculation. On day 50 post tumor inoculation, approximately 1 month after tumor eradication for the cured mice, the treated mice and naïve control mice were challenged with 2 × 10^6^ CT26 cells on the contralateral, left flank. None of the treated mice developed tumors on the left flank within 21 days (Fig. 4f), but two mice had to be euthanized due to large primary tumor burden. The three cured mice remained tumor-free 60 days after tumor challenge, indicating strong anticancer immune memory effect. In comparison, all mice in the control group consistently developed tumors after injection with 2 × 10^6^ CT26 cells (Fig. 4f).

### Antitumor immunity

**Fig. 6:**
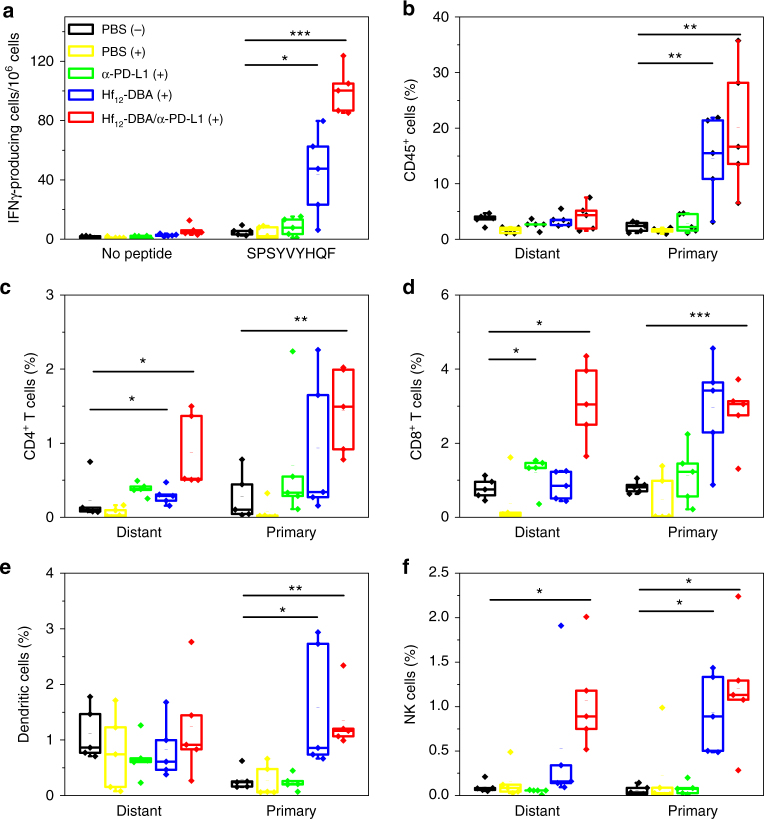
Tumor-specific immune responses. Bilateral tumor models of CT26 were established and treated as described in Fig. 4. Ten days after the first treatment, the splenocytes were harvested and stimulated with 10 μg/mL SPSYVYHQF peptide for 42 h. ELISPOT assay was performed to detect IFN-γ producing T cells (**a**). The primary (right) and distant (left) tumors were collected for flow cytometry analysis and the percentage of tumor-infiltrating CD45^+^ cells (**b**), CD4^+^ T cells (**c**), CD8^+^ T cells (**d**), dendritic cells (**e**), and NK cells (**f**) with respect to the total tumor of cells treated with PBS dark control, PBS, anti-PD-L1 antibody, Hf_12_-DBA, or Hf_12_-DBA plus anti-PD-L1 antibody with X-ray irradiation. *n* = 5. *P < 0.05 from control, **P < 0.01 from control, and ***P < 0.001 from control by *t* test. Central lines, bounds of box and whiskers represent mean values, 25% to 75% of the range of data and 1.5-fold of interquartile range away from outliers, respectively

We then used Enzyme-Linked ImmunoSpot (ELISPOT) and flow cytometry to determine the antitumor immunity of CT26 tumor-bearing mice treated with Hf_12_-DBA-mediated RT plus anti-PD-L1 antibody. The numbers of tumor-antigen-specific cytotoxic T cells were determined by a interferon-γ (IFN-γ) ELISPOT assay. At day 15 after the first treatment, we harvested splenocytes from CT26 tumor-bearing mice and stimulated them with SPSYVYHQF, a tumor-associated antigen, for 42 h. The IFN-γ spot-forming cells were then counted by Immunospot Reader. The number of antigen-specific IFN-γ-producing T cells was significantly higher in tumor-bearing mice treated with Hf_12_-DBA plus anti-PD-L1 antibody (100.2 ± 15.7) than those treated with PBS (5.3 ± 2.7) or Hf_12_-DBA (8.2 ± 6.1, Fig. 6a). These results suggest that Hf_12_-DBA with X-ray irradiation plus anti-PD-L1 antibody effectively generates tumor-specific T cell response.

We further profiled infiltrating leukocytes in both the primary and distant tumors. There was no significant difference between PBS with or without X-ray irradiation, demonstrating that low-dose X-ray irradiation did not influence the immunological environment of tumors. The Hf_12_-DBA with antibody group showed significant increase of tumor-infiltrating CD4^+^ T cells and CD8^+^ T cells in both the primary tumors and the distant tumors (Fig. 6c, d). Specifically, for the primary tumor, the percentage of CD8^+^ T cells in the total tumor cells significantly increased in both Hf_12_-DBA-mediated RT (2.92 ± 1.58 %) and Hf_12_-DBA-mediated RT plus anti-PD-L1-treated groups (2.42 ± 1.31%) compared to the PBS control group (0.67 ± 0.40%). For the distant tumor, the percentage of CD8^+^ T cells in the total tumor cells increased in Hf_12_-DBA-mediated RT plus anti-PD-L1 treatment group (2.04 ± 1.24%) compared to Hf_12_-DBA-mediated RT group (1.21 ± 0.48%) and PBS control group (1.20 ± 0.20%) (Fig. 6c). The significant increase of infiltrating CD8^+^ T cells, which was further confirmed by immunofluorescence imaging (Supplementary Method 4) as shown in Supplementary Fig. 28, likely induced the abscopal effect. The Hf_12_-DBA with antibody group showed significant increase of tumor-infiltrating B cells (Supplementary Fig. 29) as well as CD45^+^ leukocyte cells (Fig. 6b) in the primary tumors but not in the distant tumors. No significant difference was observed across the different treatment groups in the amount of CD4^+^, regulatory, and CD8^+^ T cells in the lymph nodes (Supplementary Fig. 30).

In immunotherapy, dendritic cells can be recruited to the tumor sites and then present antigens to T cells after migration to the lymph nodes. Hf_12_-DBA-mediated RT groups significantly increased the percentages of dendritic cells in primary tumors (Fig. 6e). We also found a significant increase of NK cells in both primary and distant tumors treated with Hf_12_-DBA-mediated RT and anti-PD-L1 antibody (Fig. 6f). These results suggested that the combination of nMOF-mediated RT and PD-L1 checkpoint blockade therapy not only augmented tumor-specific adaptive response but also induced innate immune response in tumors.

## Discussion

A major challenge in RT is to maximize the therapeutic effect while minimizing deleterious effects on the surrounding healthy tissues. The therapeutic ratio of RT can be enlarged with radioenhancers that can effectively increase differential radiation absorption between healthy and tumor tissues. Although NPs of high-*Z* elements, such as HfO_2_ and Au NPs, have been extensively studied for this purpose, no NP-based radioenhancer has been approved by the Food and Drug Administration for clinical use. In the study of Au NPs, it was found that ROS generation is inversely proportional to the particle diameter, indicating that larger specific surface areas may be an important design parameter.^41^ We therefore hypothesize that Hf-based nMOFs, Hf_6_-DBA and Hf_12_-DBA, which possess both high-*Z* elements and high specific surface areas, can be ideal candidates for radiation enhancement (Fig. 2b). Moreover, the porous structure of MOFs can facilitate fast diffusion of ROS, which have very short lifetimes typically on the order of 10^−7^ to 10^−4^ s.

Compared to incident X-rays, secondary photons generated from the enhanced photoelectric and/or Compton effects have lower energies and are more likely to interact with the radioenhancers. However, in the case of solid NPs, the secondary photons are only generated on the surface of NPs and cannot be effectively used since the photons are randomly scattered in all directions and have low probability to encounter other NPs. In contrast, the periodic structure of nMOFs may allow for effective use of secondary photons and electrons. Each Hf SBU is surrounded by other Hf clusters extended in all directions with inter-cluster distance typically shorter than 2 nm. As a result, there is a higher probability for the secondary photons generated inside the MOFs to interact with other metal clusters, setting off a chain reaction to enhance the overall efficacy of RT.

In order to examine the efficacy of nMOFs with high-*Z* elements for RT, we synthesized two Hf-based nMOFs of <100 nm in sizes. As a control, ultra-small solid HfO_2_ NPs with a diameter of ~10 nm were also synthesized. Normalized to the same amount of Hf, both Hf_6_-DBA and Hf_12_-DBA outperformed HfO_2_ NPs in radioenhancement, evidenced by in vitro and in vivo results. The enhanced RT effect of Hf_6_-DBA and Hf_12_-DBA may be attributed to their large specific surface areas of Hf_6_ or Hf_12_ clusters and porous framework structures. Interestingly, Hf_12_-DBA is a better radioenhancer than Hf_6_-DBA, likely due to more efficient X-ray absorption of Hf_12_ clusters over Hf_6_ clusters, leading to increased generation of hydroxyl radicals as evidenced by APF assay. To support this hypothesis, we synthesized two anthracene-based Hf-nMOFs, Hf_6_-DBAn and Hf_12_-DBAn, which are structurally similar to Hf_6_-DBA and Hf_12_-DBA, respectively, and studied their radioluminescence. Upon X-ray irradiation, Hf clusters absorb energy and subsequently transfer the energy to the anthracene-based ligands to generate luminescence. The luminescence intensity can then be used as a measure of X-ray absorption efficiency. Normalized to the same amount of Hf, Hf_12_-DBAn has fewer DBAn ligands (0.75 DBAn per Hf) than Hf_6_-DBAn (1 DBAn per Hf), but afforded much higher X-ray-induced luminescence intensity. The radioluminescence result supports the hypothesis that Hf_12_ clusters absorb X-ray more efficiently than Hf_6_ clusters.

**Fig. 7:**
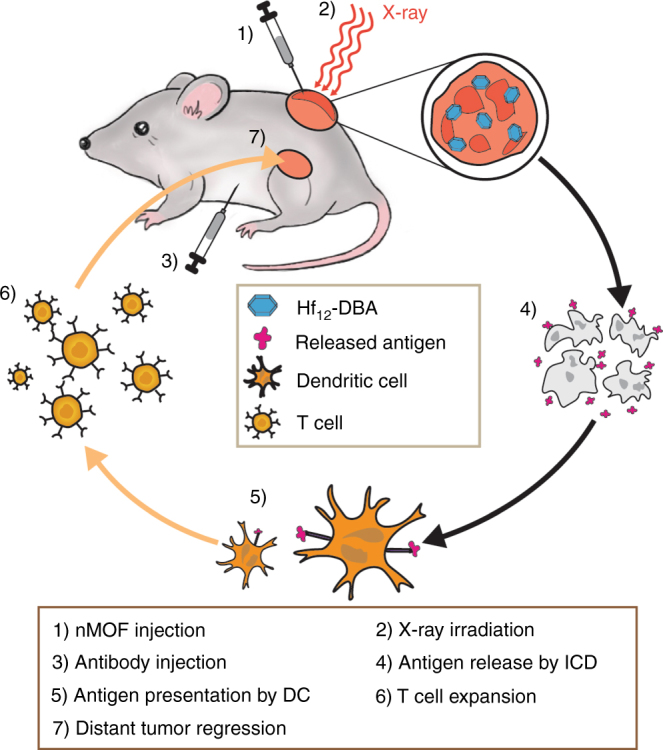
Abscopal effect of nMOF-mediated RT and immune checkpoint blockade using fractionated X-rays. nMOF was intratumorally injected into the primary tumors of mice bearing bilateral subcutaneous tumors. nMOF-mediated RT destroyed the irradiated tumors, caused immunogenic cell death, and released tumor antigen. Injected anti-PD-L1 antibody overcame the suppressive tumor microenvironment by targeting PD-1/PD-L1 axis. The combination of nMOF-mediated RT and anti-PD-L1 checkpoint blockade led to the effective T cell expansion and tumor infiltration, which effectively suppressed/eradicated the distant tumors

As a local therapy, hypofractionated RT with relatively high daily doses of 5–10 Gy and a shortened treatment duration can also trigger a local immune response by releasing immunostimulating signals to increase T cell infiltration to the tumor.^42–45^ Over one hundred of clinical trials are ongoing to exploit the synergy between hypofractionated RT and immune checkpoint blockade.^17,46–48^ However, the immunostimulatory effects of hypofractionated RT are achieved at the expense of damaging side effects on surrounding tissues.^49^ Furthermore, the dosing schedule of hypofractionated RT is not synchronous with checkpoint blockade immunotherapy.^50^ We hypothesize that effective radioenhancers can not only enhance the localized therapeutic effects of RT but can also lead to immunostimulatory tumor microenvironments that can potentiate checkpoint blockade immunotherapy. In this study, we showed that Hf_12_-DBA not only exhibited superior radioenhancing effects to improve regression of irradiated tumors as local therapy but also regressed distant, un-irradiated tumors when used in combination with an anti-PD-L1 antibody (Fig. 7). The combination of nMOF-mediated RT with an anti-PD-L1 antibody thus effectively extended the local therapeutic effects of RT to distant tumors via abscopal effects, affording a potent systemic antitumor treatment.

PD-1 is a cell-surface co-inhibitory receptor expressed on T cells, B cells, monocytes, and NK cells with two known ligands, PD-L1 and PD-L2.^51^ PD-L1 is upregulated on tumor cells and other cells in the tumor microenvironment. Blockade of the PD-1/PD-L1 axis using anti-PD-1 or anti-PD-L1 antibodies can restore T cell activity against tumor cells, providing potent antitumor efficacy. Infiltrating T cells are essential for PD-L1 blockade therapy to be effective but are only found in immunogenic tumor microenvironments. As a result, checkpoint blockade immunotherapy is only effective in patients whose tumors are immunogenic, leading to the low rate of durable responses in many cancers. Our work shows that nMOF-mediated RT provides an efficient way to induce immunogenicity in the tumor microenvironment and enhance antitumor immunity of anti-PD-L1. We have elucidated the mechanisms behind nMOF-mediated radioenhancement and the immunotherapeutic effect of combination treatment with Hf_12_-DBA and irradiation plus an anti-PD-L1 antibody. The numbers of antigen-specific IFN-γ-producing T cells and CD8^+^ T cells significantly increased in distant, un-irradiated tumors to lead to tumor rejection/regression. It is possible that nMOF-mediated RT can be combined with other forms of immunotherapies such as STING agonists or CpG oligonucleotides to lead to systemic antitumor immunity.

In summary, we have developed Hf-based nMOFs as effective radioenhancers for low-dose X-ray RT. The combination of nMOF-mediated RT and PD-L1 checkpoint blockade extended the local therapeutic effects of RT to distant tumors via systemic antitumor immunity. With the ability to rationally design and systematically tune the compositions and structures of nMOFs, we expect that even more powerful nMOF-based radioenhancers will become available to drastically enhance the efficacy of RT without incurring deliberating side effects and to significantly potentiate checkpoint blockade immunotherapy for the treatment of non-immunogenic tumors.

## Methods

### Cell lines and animals

Human head and neck cancer cells, SQ20B and JSQ3, and murine breast cancer cells, TUBO and 4T1, were kindly provided by Dr. Stephen J. Kron at the University of Chicago. The murine colon adenocarcinoma cell, CT26, and the human cervical cancer cell, HeLa, were purchased from the American Type Culture Collection (Rockville, MD, USA). TUBO and CT26 cells were cultured in Roswell Park Memorial Institute (RPMI) 1640 medium (GE Healthcare, USA) supplemented with 10% fetal bovine serum (FBS, VWR, USA). 4T1 and HeLa cells were cultured in Dulbecco’s modified Eagle’s medium (DMEM) (GE Healthcare, USA) supplemented with 10% FBS. SQ20B and JSQ3 cells were cultured in DMEM and Ham’s F-12 nutrient mixture (DME/F12) medium (GE Healthcare, USA) supplemented with 20% FBS. All medium was further supplemented with 100 U/mL penicillin G sodium and 100 μg/mL streptomycin sulfate. Cells were cultured in a humidified atmosphere containing 5% CO_2_ at 37 °C. Mycoplasma was tested before use by MycoAlert Detection Kit (Lonza Nottingham Ltd.) BALB/c mice (6–8 weeks) were obtained from Harlan-Envigo Laboratories Inc. (USA). The study protocol was reviewed and approved by the Institutional Animal Care and Use Committee (IACUC) at the University of Chicago.

### Synthesis of Hf_6_-DBA and Hf_6_-DBAn nMOFs

To a 1 dram glass vial was added 0.5 mL of HfCl_4_ solution (2.0 mg/mL in dimethylformamide (DMF)), 0.5 mL of the 2,5-di(*p*-benzoato)aniline (H_2_DBA) solution (2.0 mg/mL in DMF) or 2,5-di(*p*-benzoato)anthracene (H_2_DBAn) solution (2.5 mg/mL in DMF) and 0.5 μL of trifluoroacetic acid. The reaction mixture was kept in a 60 °C oven for 72 h. The white precipitate was collected by centrifugation and washed with DMF, 1% trimethylamine/ethanol solution, and ethanol.

### Synthesis of Hf_12_-DBA and Hf_12_-DBAn nMOFs

To a 1 dram glass vial was added 0.5 mL of HfCl_4_ solution (1.6 mg/mL in DMF), 0.5 mL of the H_2_DBA solution (1.6 mg/mL in DMF) or H_2_DBAn solution (2 mg/mL in DMF), 75 μL of acetic acid, and 5 μL of water. The reaction mixture was kept in an 80 °C oven for 72 h. The white precipitate was collected by centrifugation and washed with DMF, a 1% trimethylamine/ethanol solution, and ethanol.

### Synthesis of HfO_2_ NPs

HfO_2_ NPs were synthesized according to the reported protocol.^52^ To a 20 mL Teflon cup was added 15 mL of HfCl_4_ solution (10 mg/mL in benzyl alcohol), which was sealed in a steel autoclave and heated to 200 °C for 48 h. The white precipitate was collected by centrifugation and washed with ethanol.

### Radioluminescence measurements of Hf_12_-DBAn and Hf_6_-DBAn

Hf_12_-DBAn and Hf_6_-DBAn were suspended in ethanol at an Hf concentration of 1 mM, degassed, and refilled with nitrogen gas. The solutions were transferred to 1 dram vials for X-ray irradiation with a maximum dose rate of 2.93 Gy/min. X-ray luminescence measurements were acquired with a small animal irradiator (X-RAD 225Cx by Precision X-ray) through a 2 mm aluminum filter. The radioluminescence was detected using a cooled CoolSNAP HQ2 CCD camera (Photometrics, USA) equipped with a DX Micro-NIKKOR f/2.8 macro lens (Nikon, Japan). Samples were tested at a voltage of 70 kVp and current values ranging from 5, 10, 20, 30 to 40 mA for 30 s. Radioluminescence intensities were processed by ImageJ with background subtraction.

### Detection of hydroxyl radical produced by irradiation

HfO_2_, Hf_6_-DBA, and Hf_12_-DBA were suspended in water at equivalent Hf concentrations of 20 μM in the presence of 5 μM APF. A water solution of 5 μM APF was used as background. One hundred microliters of each suspension was added to a 96-well plate and then irradiated with 0, 1, 2, 3, 5, or 10 Gy X-ray (Philips RT250 X-ray generator, Philips, USA, 250 KVp, 15 mA, 1 mm Cu filter). The fluorescence signal was immediately collected with a Xenogen IVIS 200 imaging system.

### Clonogenic assay

The clonogenic assay was performed according to a modified protocol.^53^ CT26 cells were cultured in a 6-well plate overnight and incubated with particles at an Hf concentration of 20 µM for 4 h followed by irradiation with 0, 1, 2, 4, 8, and 16 Gy X-ray (250 kVp, 15 mA, 1 mm Cu filter) or γ-ray (^60^Co source, Atomic Energy Canada Limited, Canada). Cells were trypsinized and counted immediately. One hundred to one thousand cells were seeded in a 6-well plate and cultured with 2 mL medium for 10–20 days. Once colony formation was observed, the culture medium was discarded. The plates were rinsed twice with PBS, and then stained with 500 µL of 0.5% w/v crystal violet in 50% methanol/H_2_O. The wells were rinsed with water and the colonies were counted manually.

### Apoptosis/necrosis

CT26 cells were cultured in a 6-well plate overnight and incubated with particles at an Hf concentration of 20 µM for 4 h followed by irradiation with 0 or 2 Gy X-ray (250 kVp, 15 mA, 1 mm Cu filter). After 24 h, the cells were stained according to the AlexaFluor 488 Annexin V/Dead Cell Apoptosis Kit (Life Technology, USA) and quantified by flow cytometry.

### DNA damage

CT26 cells were cultured in a 6-well plate overnight and incubated with particles at an Hf concentration of 20 µM for 4 h followed by irradiation at 0 and 2 Gy X-ray (250 kVp, 15 mA, 1 mm Cu filter). Cells were stained immediately with the HCS DNA Damage Kit (Life Technology, USA) for CLSM (FV1000, Olympus, Japan) and flow cytometry. ImageJ was used to quantify the number of cells with foci and number of intranuclear foci.

### Immunogenic cell death

CT26 cells were cultured in a 6-well plate overnight and incubated with particles at an Hf concentration of 20 µM for 4 h followed by irradiation with 0 or 2 Gy X-ray (250 kVp, 15 mA, 1 mm Cu filter). After incubation for 4 h, the cells were washed three times with PBS, fixed with 4% paraformaldehyde, incubated with AlexaFluor 488-CRT (Enzo Life Sciences, USA) with 1:100 dilution for 2 h, stained with 4′,6-diamidino-2-phenylindole, and observed by CLSM. Treated cells were incubated for 4 h, collected, incubated with AlexaFluor 488-CRT antibody for 2 h, and then stained with propidium iodide (PI) for analysis by flow cytometry (LSRFortessa, BD, USA).

### In vivo anticancer efficacy

A bilateral model was established by subcutaneously inoculating 2 × 10^6^ and 1 × 10^6^ CT26 cells onto the right and left flanks of BALB/c mice for respective primary and secondary tumors. When the primary tumors reached 100–150 mm^3^ in volume, mice were injected intratumorally with nMOFs at a dose of 1 µmol Hf or PBS. Twelve hours after injection, mice were anesthetized with 2% (v/v) isoflurane and the primary tumors were irradiated with 1 Gy X-ray/fraction (120 kVp, 20 mA, 2 mm Cu filter) for a total of 10 daily fractions. Anti-PD-L1 antibody was given every 3 days by intraperitoneal injection at a dose of 75 µg/mouse. The tumor sizes were measured daily with a caliper where tumor volume equals (width^2^ × length)/2. Mice treated with Hf_6_-DBA and Hf_12_-DBA were sacrificed on day 30 and mice treated with PBS or anti-PD-L1 antibody alone were sacrificed on day 21. Each mouse was weighed daily to evaluate toxicity.

### T cell depletion

The bilateral subcutaneous model was established as for the in vivo anticancer efficacy. When the primary tumors reached 100–150 mm^3^ in volume, mice were injected intratumorally with nMOFs at a dose of 1 µmol Hf or PBS. Anti-CD4 (GK1.5, BioXCell, USA), anti-CD8 (OKT-8, BioXCell, USA), or mouse IgG (C1.18.4, BioXCell, USA) antibodies were intraperitoneally injected into the mice (200 µg/mouse per injection) on days 0 and 5 after the first treatment. Ten hours post injection, mice were anesthetized with 2% (v/v) isoflurane, and tumors were irradiated with image-guided X-ray at 225 kVp and 13 mA with a 0.3 mm Cu filter. To evaluate the therapeutic efficacy, the tumor growth and body weight were monitored daily.

### Tumor challenge studies

CT26 cells (2 × 10^6^) were inoculated subcutaneously onto the right flank of BALB/c mice. When the tumors reached 100–150 mm^3^ in volume, mice were injected intratumorally with nMOFs at a dose of 1 µmol Hf or PBS. Twelve hours after injection, mice were anesthetized with 2% (v/v) isoflurane and the primary tumors were irradiated with 1 Gy X-ray/fraction (120 kVp, 20 mA, 2 mm Cu filter) for a total of 10 daily fractions. On day 50 post inoculation, mice were challenged with 2 × 10^6^ cells on the contralateral flank. Healthy mice were simultaneously inoculated as control. The mice were sacrificed when the tumors of the control mice reached 2 cm^3^. Statistical analysis was performed using the log-rank Kaplan–Meier estimation.

### ELISpot assay

ELISpot assay (Mouse IFN-γ ELISpot Ready-SET-Go!; Cat. No. 88-7384-88; eBioscience) was used to determine tumor-specific immune responses to IFN-γ in vitro. A Millipore Multiscreen HTS-IP plate was coated with anti-mouse IFN-γ capture antibody at 4 °C overnight. Single-cell suspensions of splenocytes were obtained from CT26 tumor-bearing mice and seeded onto the antibody-coated plate at a concentration of 2 × 10^5^ cells/well. Cells were incubated with or without SPSYVYHQF stimulation (10 µg/ml; in purity >95%; PEPTIDE 2.0) for 42 h at 37 °C and then discarded. The plate was then incubated with biotin-conjugated anti-IFN-γ detection antibody at room temperature (r.t.) for 2 h, followed by incubation with Avidin-HRP at r.t. for 2 h. A solution of 3-amino-9-ethylcarbazole (Sigma, Cat. No. AEC101) was added as a substrate for cytokine spot detection. Spots were imaged and quantified with a CTL ImmunoSpot Analyzer (Cellular Technology Ltd, USA).

### Lymphocyte profiling

Tumors were harvested, and then treated with 1 mg/ml collagenase I (Gibco, USA) for 1 h at 37 °C. Cells were filtered through nylon mesh filters with a size of 40 µm and washed with PBS. Tumor-draining lymph nodes were collected and directly ground through the cell strainers. The single-cell suspension was incubated with anti-CD16/32 (clone 93) to reduce nonspecific binding to FcRs. Cells were further stained with the following fluorochrome-conjugated antibodies: CD45 (30-F11), CD3ε (145-2C11), CD4 (GK1.5), CD8 (53-6.7), Foxp3 (FJK-16s), CD25 (PC61.5), Nkp46 (29A1.4), F4/80 (BM8), B220 (RA3-6B2), and PI (all from eBioscience). Antibodies were used at a dilution of 1:200. Representative gating strategies for different immune cells are shown in Supplementary Fig. 31. LSRFortessa (BD Biosciences) was used for cell acquisition and data analysis was carried out with the FlowJo software (Tree Star, Ashland, OR, USA).

### Data availability

The authors declare that all the data supporting the findings of this study are available within the article and its Supplementary Information files or from the corresponding author upon reasonable request.

## Electronic supplementary material

Supplementary Information
