# Supplementary material for: Nanoscale metal-organic frameworks enhance radiotherapy to potentiate checkpoint blockade immunotherapy
Source: Nat Commun. 2018 Jun 15;9:2351. doi: 10.1038/s41467-018-04703-w (PMC6003951; doi:10.1038/s41467-018-04703-w)
Supplement: Supplementary file 1 — Supplementary Information [file 41467_2018_4703_MOESM1_ESM.pdf]

Supplementary Information

Nanoscale Metal-Organic Frameworks Enhance Radiotherapy to Potentiate Checkpoint Blockade Immunotherapy

Kaiyuan Ni[1,†], Guangxu Lan[1,†], Christina Chan[1], Bryan Quigley[2], Kuangda Lu[1,3], Theint Aung[1], Nining Guo[1,3], Patrick La Riviere[2], Ralph R. Weichselbaum[3], Wenbin Lin[1,3*]

[1]Department of Chemistry, The University of Chicago, Chicago, IL 60637, USA.

[2]Department of Radiology, The University of Chicago, Chicago, IL 60637, USA.

[3]Department of Radiation and Cellular Oncology and The Ludwig Center for Metastasis Research, The University of Chicago, Chicago, IL 60637, USA.

[*]Corresponding author. E-mail: wenbinlin@uchicago.edu

[†]These authors contributed equally to this work.

Supplementary Methods

Supplementary Method 1: Detection of hydroxyl radical produced by Fenton's reaction

HfO_2, Hf_6-DBA, and Hf_{12}-DBA were suspended in water at equivalent Hf concentrations of 20 μM. Aqueous solutions of 5 μM APF, 50 μM $FeCl_2$, and various H_2O_2 concentration of 0, 100, 200, 400, 800, 1600, 3200, and 6400 μM were separately prepared. 100 μL of nanoparticle suspension and aqueous solution of APF, $FeCl_2$, and H_2O_2 were added to a 96-well plate and the fluorescence signal was immediately collected with a Xenogen IVIS 200 imaging system (Xenogen, USA). Nanoparticle-free aqueous solutions of APF, $FeCl_2$, and H_2O_2 served as controls.

Supplementary Method 2: Cellular uptake

The cellular uptake of Hf_6-DBA, Hf_{12}-DBA, and HfO_2 nanoparticles was evaluated in CT26 cells. Cells were seeded on 6-well plate at 1×10^6/well and further cultured for 12 h. Particles were added to the cells at a Hf concentration of 20 μM. After incubation of 1, 2, 4 and 8 hours, the cells were collected and the cell numbers were counted by a hemocytometer. Cells were digested with 1% hydrofluoric acid and concentrated nitric acid in a microwave reactor (CEM, USA) and the Hf concentrations were determined by ICP-MS (Agilent, USA). Results were expressed as the amount of Hf (nmol) per 10^5 cells.

Supplementary Method 3: Endocytosis

Hf_6-DBA and Hf_{12}-DBA nMOFs were dispersed in DMF (1 mL, 1 mmol/L by DBA concentration). To the dispersions rhodamine B isothiocyanate solutions (2 mmol/L in DMF, 25 μL) were added. The mixtures were stirred in dark overnight and the resultant Hf_6-DBA-R or Hf_{12}-DBA-R nMOFs was washed with ethanol and water sequentially.

CT26 cells were seeded on a 6-well plate at 5×10^5 cells per well and cultured for 24 h. To each well 40 μmol (by total DBA content) of Hf_6-DBA-R or Hf_{12}-DBA-R was added and the cells were incubated for 4 h. The cell culture medium was removed and the cells were washed with PBS and stained with lysotracker, fixed with paraformaldehyde, and stained with DAPI in sequence. Efficient cellular uptake of nanoparticles was directly observed with confocal laser scanning microscopy (FV1000, Olympus, Japan).

Supplementary Method 4: Immunofluorescence assay

Tumors were collected and frozen tissue sections with thickness of 6 μm were prepared using a cryostat. These sections were air-dried for at least 1 h and then fixed in acetone for 10 min at 20 °C. After blocked with 20% donkey serum, the sections were incubated with individual primary antibodies against CD8 (Thermo Scientific) overnight at 4°C, followed by incubation with dye-conjugated secondary antibodies for 1 h at r.t. After stained with DAPI for another 10 min, the sections were then washed twice with PBS and observed under CLSM.

Supplementary Figures

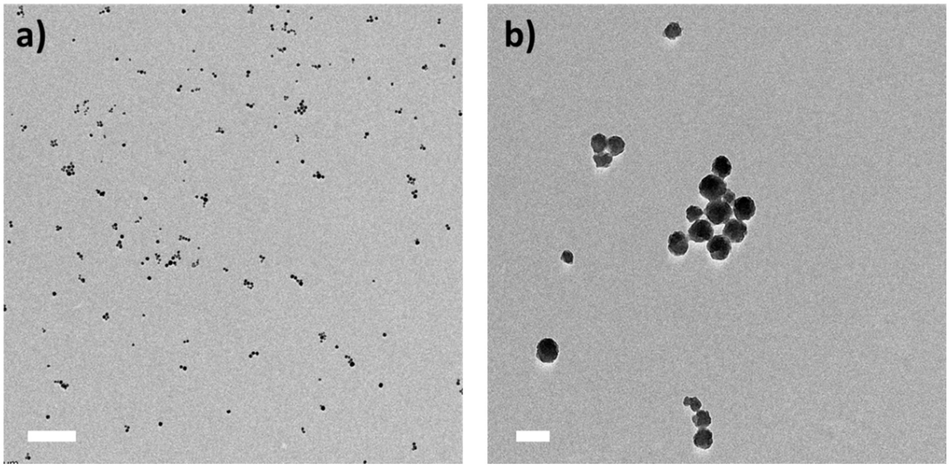

Supplementary Figure 1 TEM images of Hf_6-DBA. Scale bar = 1000 nm (a) or 100 nm (b). One of more than five repetitions with similar results is shown.

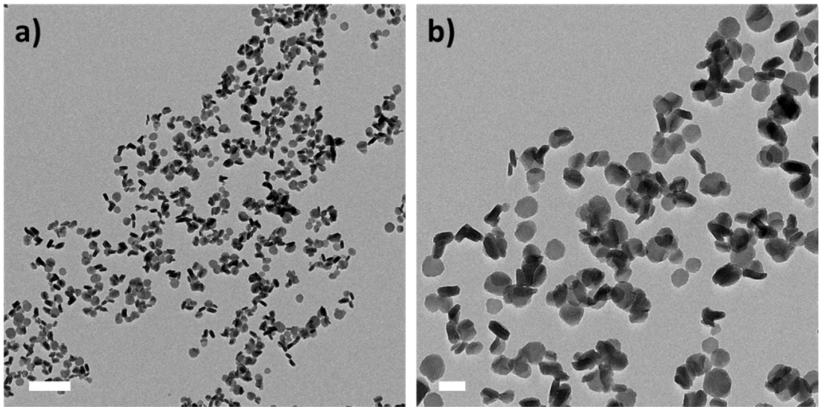

Supplementary Figure 2 TEM images of Hf_{12}-DBA. Scale bar = 500 nm (a) or 100 nm (b). One of more than five repetitions with similar results is shown.

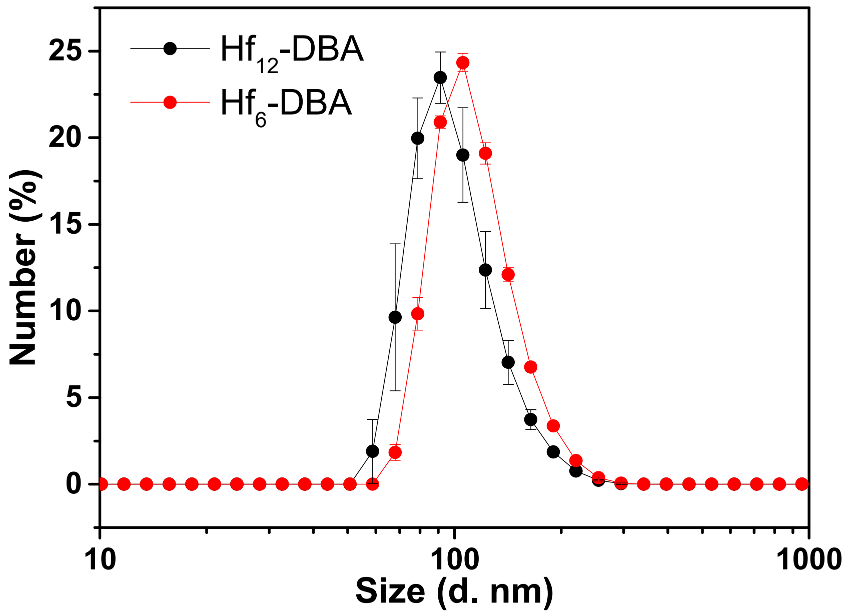

Supplementary Figure 3 Hydrodynamic sizes of Hf_6-DBA and Hf_{12}-DBA in water by dynamic light scattering (DLS) measurements. n=3. One of two repetitions with similar results is shown. Central data points and error bars represent mean ± s.d. values, respectively.

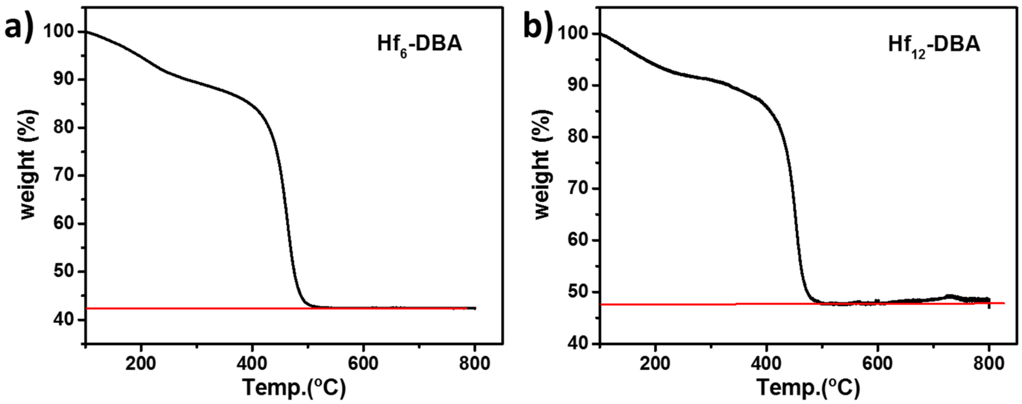

Supplementary Figure 4 TGA curves of freshly prepared Hf_6-DBA (a) and Hf_{12}-DBA (b) in the 100 ~ 800 ºC range. The weight loss corresponds to the decomposition of Hf_6-DBA or Hf_{12}-DBA to HfO_2. The weight loss of Hf_6-DBA is 58.5%, which is consistent with a calculated weight loss of 60.4% based on the conversion of $Hf_6(\mu_3\text{-}O)_4(\mu_3\text{-}OH)_4(DBA)_6$ to $(HfO_2)_6$. The weight loss of Hf_{12}-DBA is 52.3%, which is consistent with a calculated weight loss of 53.9% based on the conversion of $Hf_{12}(\mu_3\text{-}O)_8(\mu_3\text{-}OH)_8(\mu_2\text{-}OH)_6(DBA)_9$ to $(HfO_2)_{12}$. The TGA results were obtained without repetition.

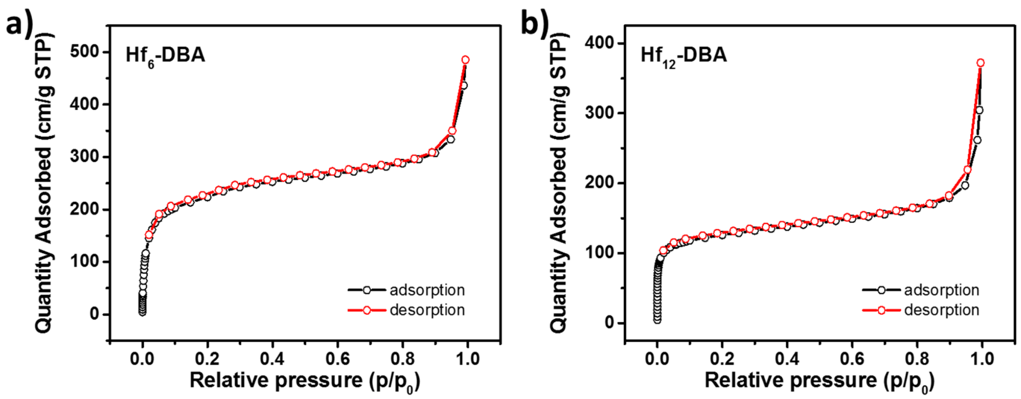

Supplementary Figure 5 Nitrogen sorption isotherms (77 K) of Hf_6-DBA (a) and Hf_{12}-DBA (b). Hf_6-DBA has a BET surface area of 804.4 m^2/g and Hf_{12}-DBA has a BET surface area of 463.9 m^2/g. The BET results were obtained without repetition.

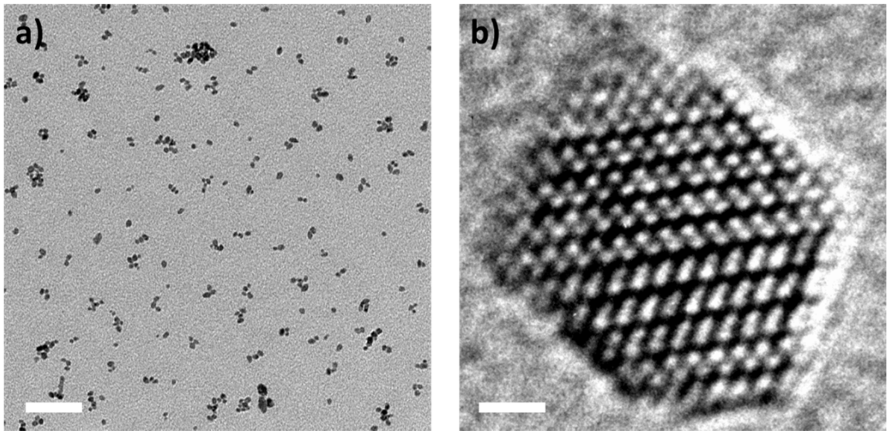

Supplementary Figure 6 TEM image (a) and high-resolution TEM image (b) of HfO_2. Scale bar = 50 nm (a) or 1 nm (b). One of three repetitions with similar results is shown.

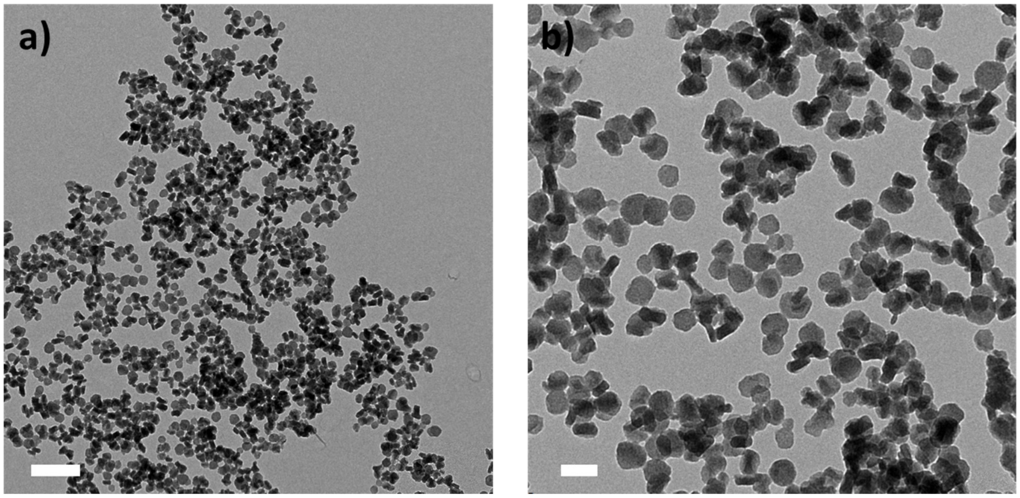

Supplementary Figure 7 TEM images of Hf_{12}-DBAafter 72-h incubation in RPMI-1640 medium. Scale bar = 500 nm (a) or 100 nm (b). One of two repetitions with similar results is shown.

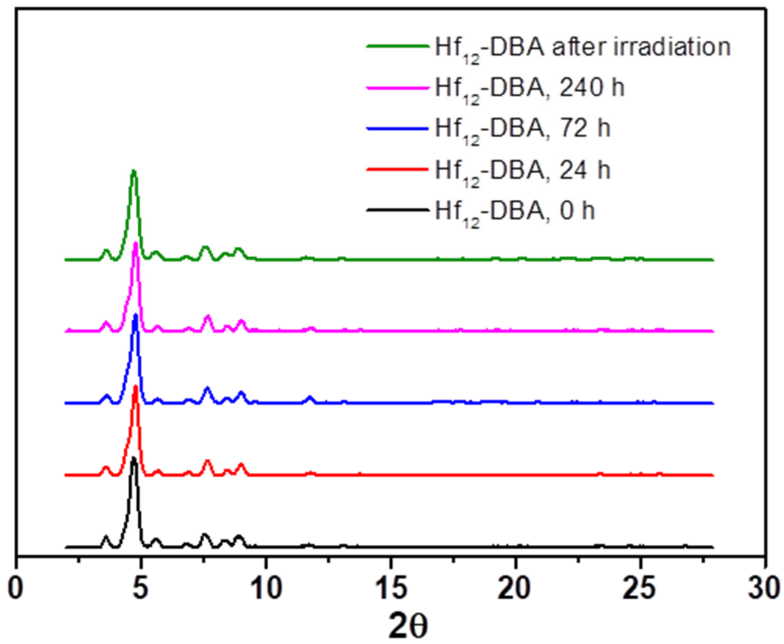

Supplementary Figure 8 PXRD patterns of Hf_{12}-DBA after 24, 72 and 240 h incubation in RPMI-1640 medium or after X-ray irradiation at a dose of 16 Gy. The PXRD results were obtained without repetition.

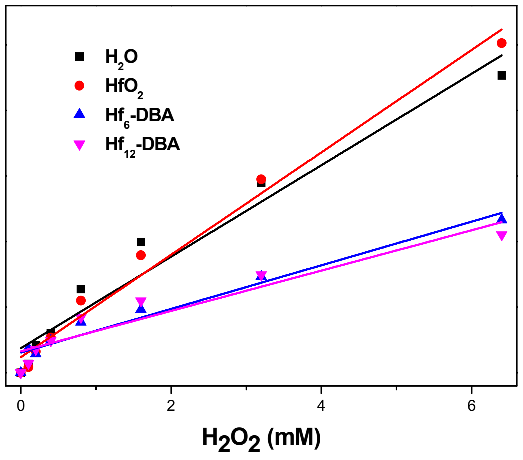

Supplementary Figure 9 APF fluorescence of H_2O, HfO_2, Hf_6-DBA, and Hf_{12}-DBA with Fenton reaction at equivalent Hf concentrations of 20 μM. The ratio of fitting slope of HfO_2, Hf_6-DBA, or Hf_{12}-DBA to H_2O is 1.12, 0.48, or 0.44, respectively. The result was obtained without repetition.

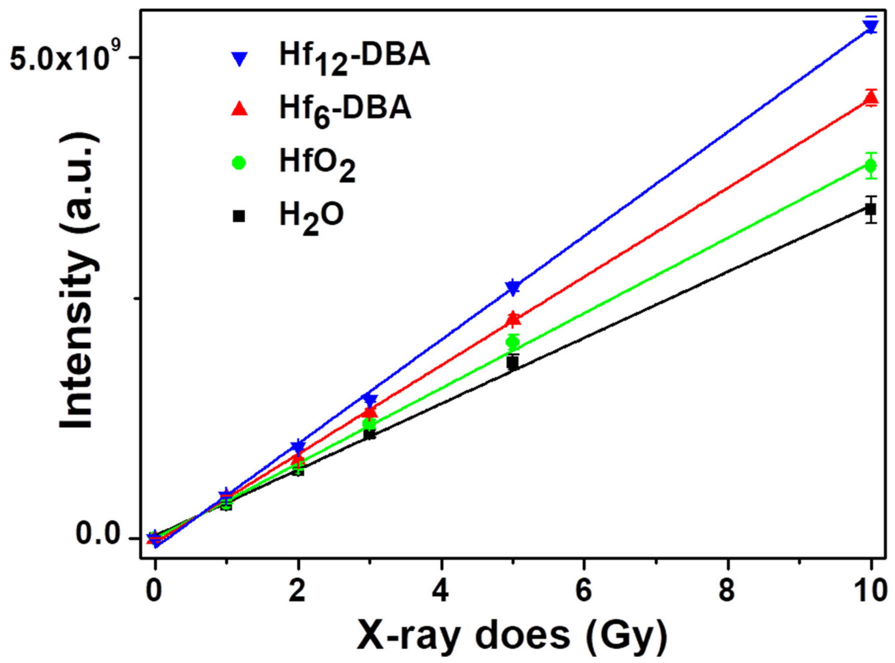

Supplementary Figure 10 APF fluorescence of H_2O, HfO_2, Hf_6-DBA, and Hf_{12}-DBA upon X-ray irradiation at equivalent Hf concentrations of 20 μM. n=6. The result was obtained without repetition. Central data points and error bars represent mean ± s.d. values, respectively.

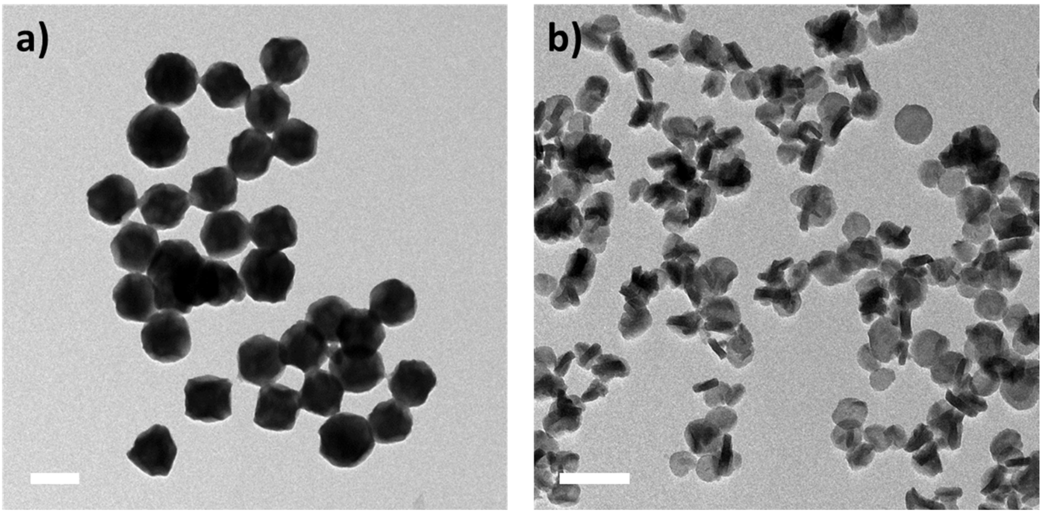

Supplementary Figure 11 TEM images of Hf_6-DBAn (a) and Hf_{12}-DBAn (b). Scale bar = 500 nm (a) or 200 nm (b). One of two repetitions with similar results is shown.

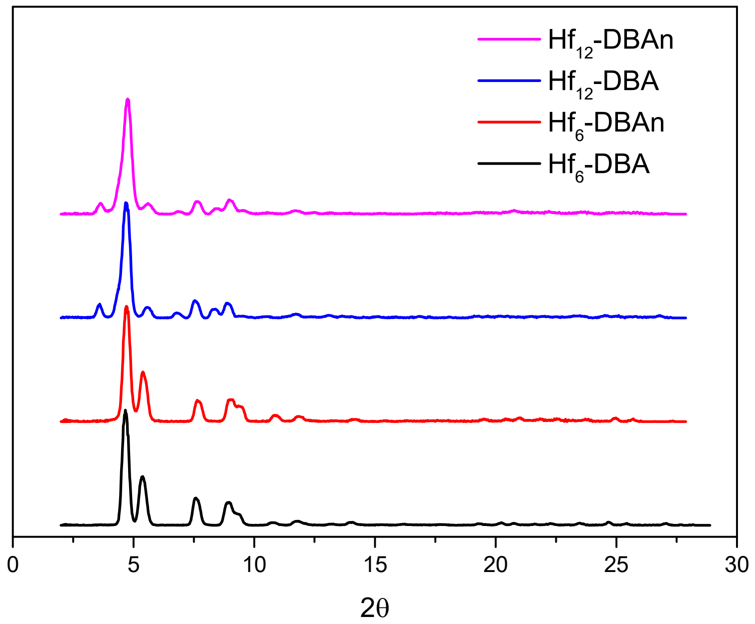

Supplementary Figure 12 PXRD patterns of Hf_6-BDA, Hf_6-DBAn, Hf_{12}-DBA and Hf_{12}-DBAn. One of two repetitions with similar results is shown.

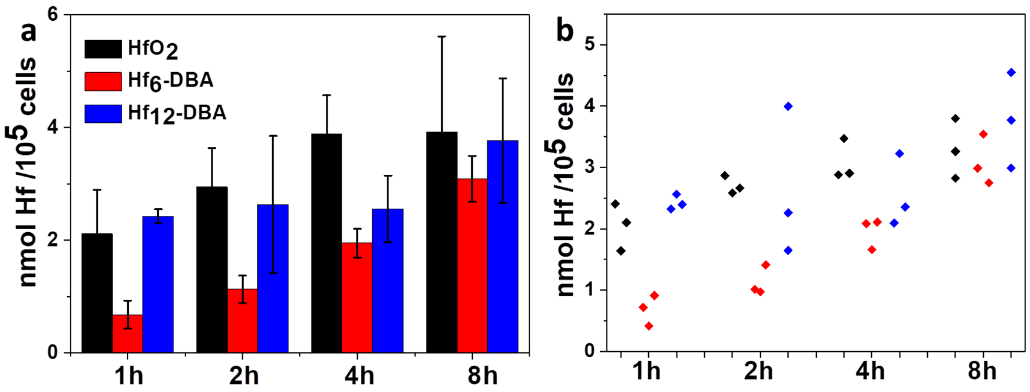

Supplementary Figure 13 Cellular uptake of three Hf-based nanoparticles after 1, 2, 4 or 8 hour incubation with equivalent Hf concentrations of 20 μM presented in (a) bar chart and (b) dot plot. The Hf concentrations were determined by ICP-MS. n = 3. One of two repetitions with similar results is shown. Central data points and error bars in (a) represent mean ± s.d. values, respectively.

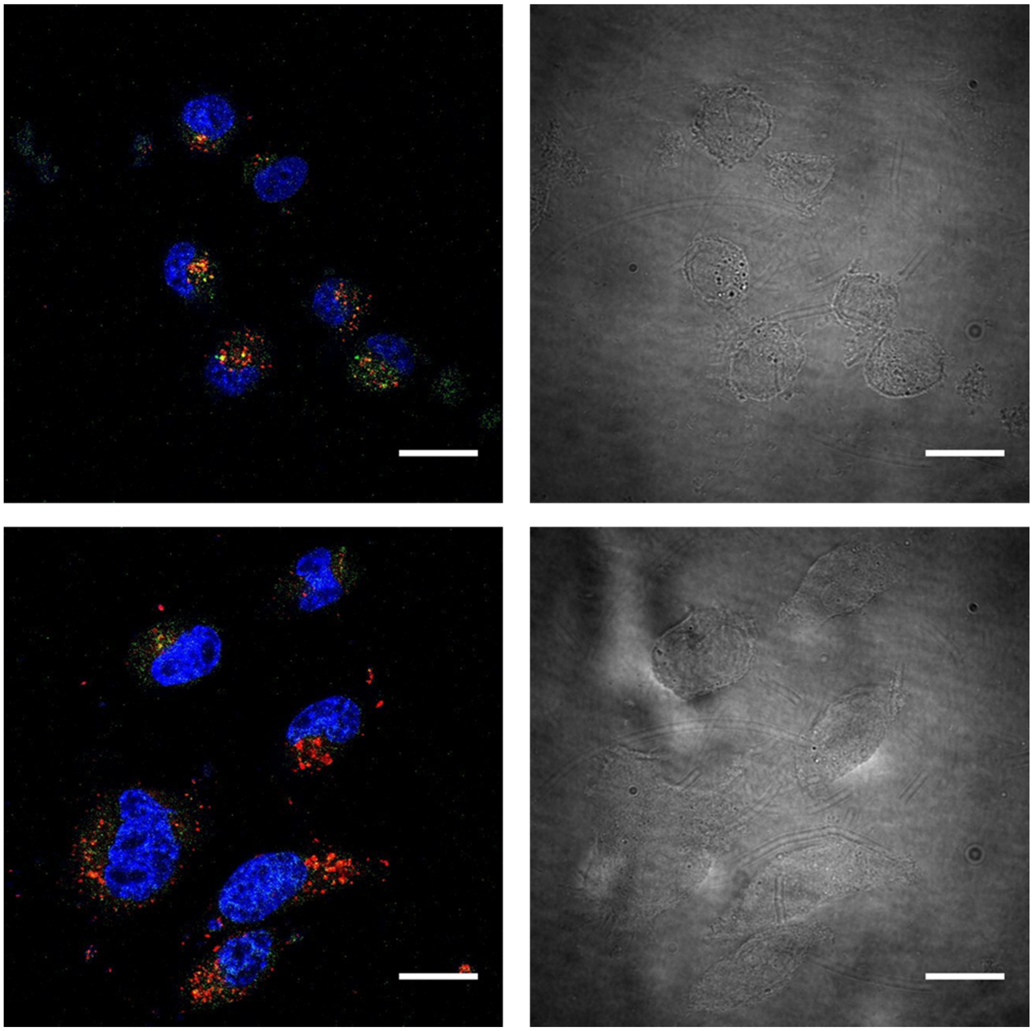

Supplementary Figure 14 Endocytosis of Hf-based nMOFs. CT26 Cells were treated with Hf_6-DBA or Hf_{12}-DBA preloaded with Rhodamine B for 4 hours. Endosome/lysosome and nuclei were stained with Lysotracker Green and DAPI, respectively. Scale bar = 10 μm. One of three repetitions with similar results is shown.

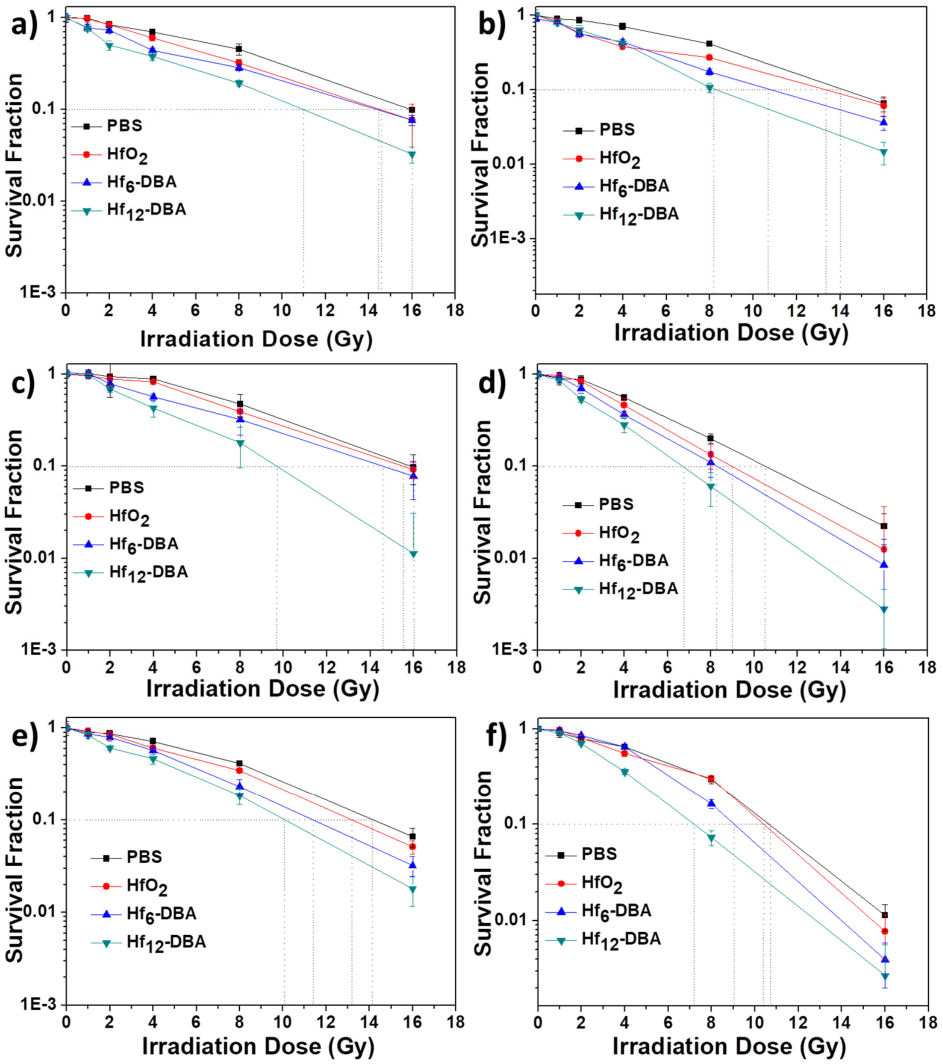

Supplementary Figure 15 Clonogenic assay for evaluating radioenhancement upon X-ray irradiation on (a) 4T1, (b) TUBO, (c) SQ20B, (d) JSQ3, (e) CT26, (f) Hela cancer cell lines. n = 6. One of two repetitions with similar results is shown. Central data points and error bars represent mean ± s.d. values, respectively.

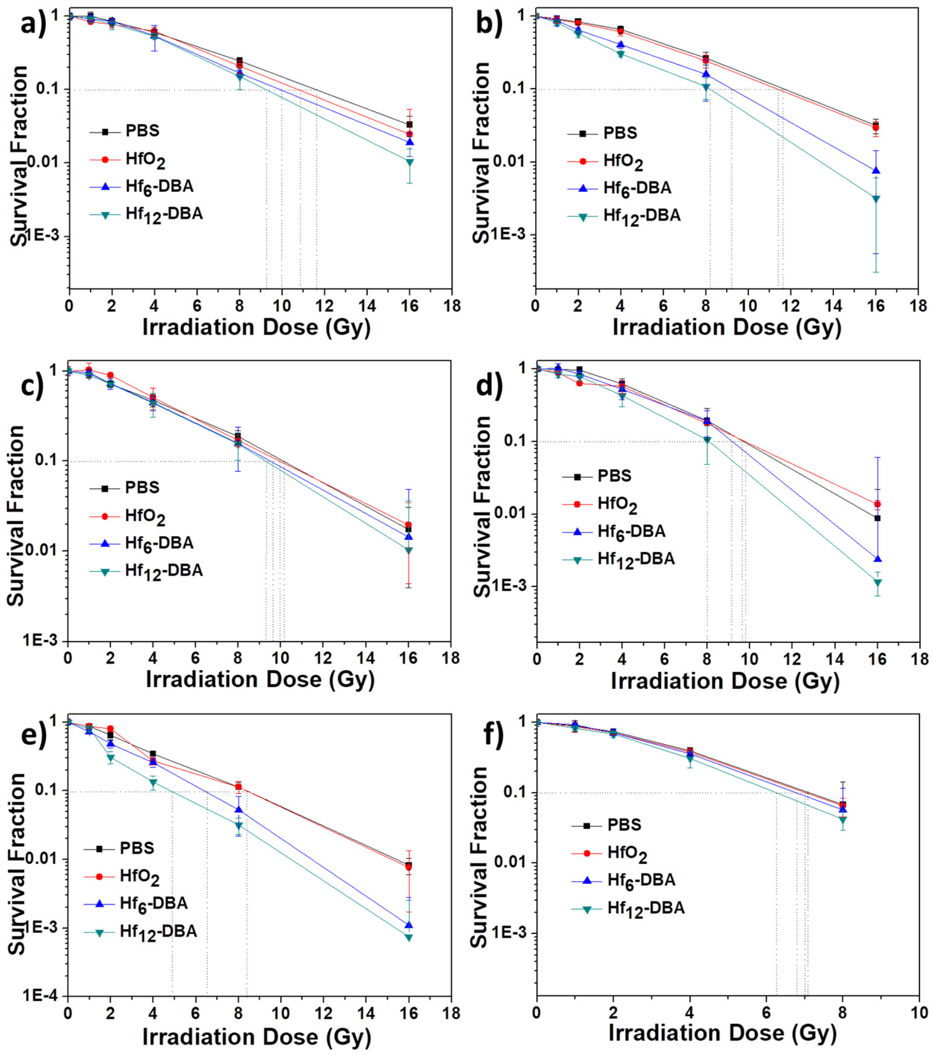

Supplementary Figure 16 Clonogenic assay for evaluating radioenhancement upon γ-ray irradiation with ^{60}Co source on (a) 4T1, (b) TUBO, (c) SQ20B, (d) JSQ3, (e) CT26, (f) Hela cancer cell lines. n = 6. One of two repetitions with similar results is shown. Central data points and error bars represent mean ± s.d. values, respectively.

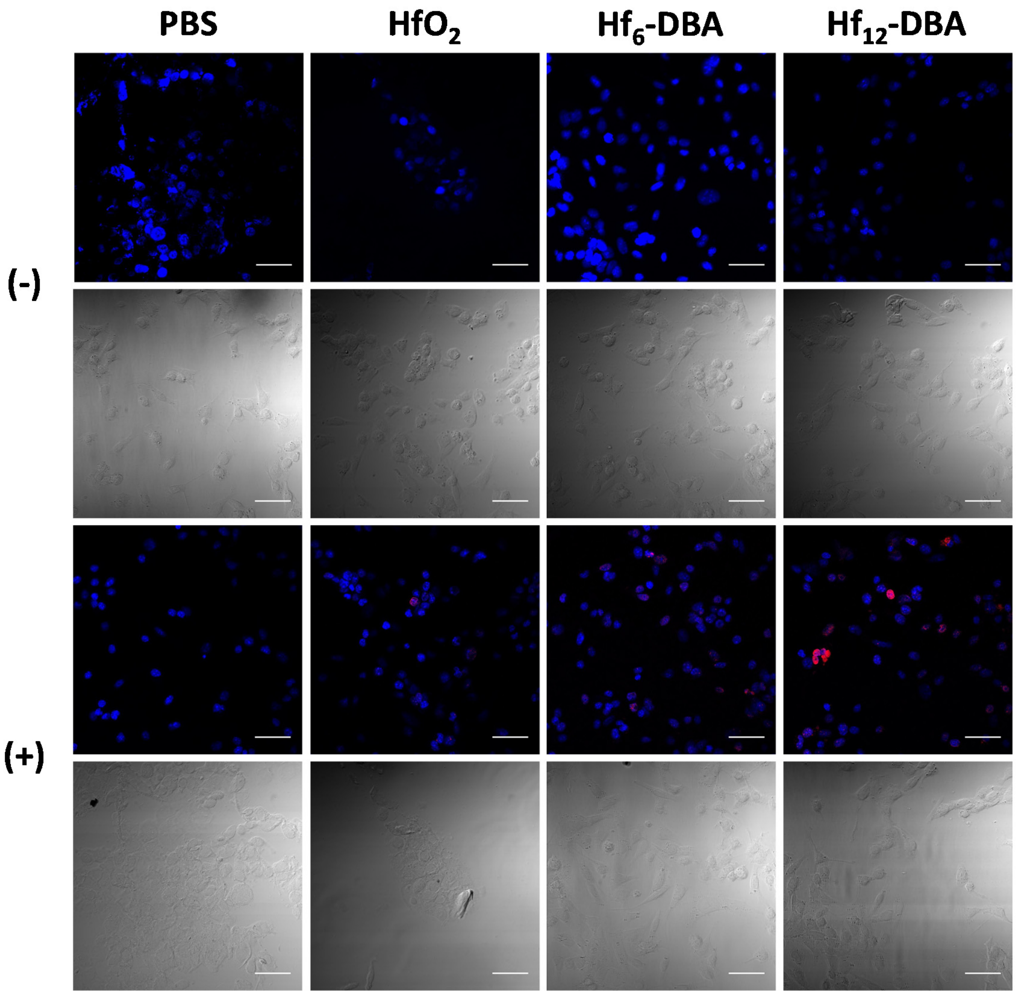

Supplementary Figure 17 Representative γ-H2AX immunostaining assay showing DNA double strain breaks (DSBs) in CT26 cells. Cells were treated with PBS or three Hf-based nanoparticles, with (+) or without (-) X-ray irradiation. Blue and red fluorescence show DAPI-stained nucleus and antibody-labeled γ-H2AX in the cells, respectively. From left to right: PBS control, HfO_2, Hf_6-DBA or Hf_{12}-DBA, respectively. Scale bar = 20 μm. One of two repetitions with similar results is shown.

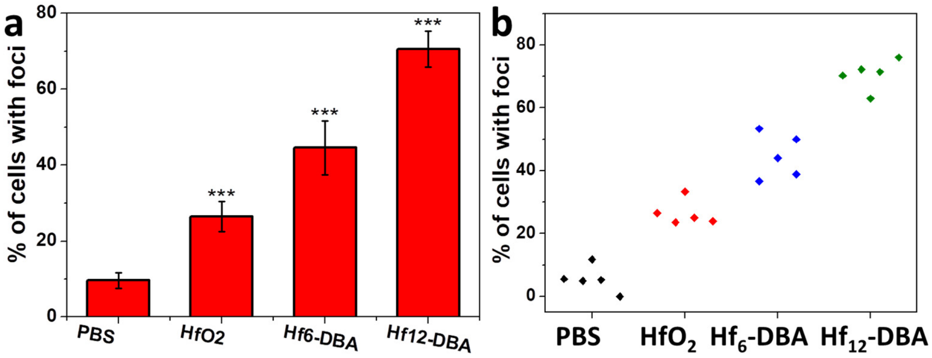

Supplementary Figure 18 The percentage of cells with nuclear foci was quantified based on γ-H2AX immunostaining assays as shown in Supplementary Figure 19 presented in (a) bar chart and (b) dot plot. Five images were collected for each group and cells with or without nuclear foci were counted for analysis. From left to right: PBS control, HfO_2, Hf_6-DBA or Hf_{12}-DBA, respectively. Central data points and error bars in (a) represent mean ± s.d. values, respectively.

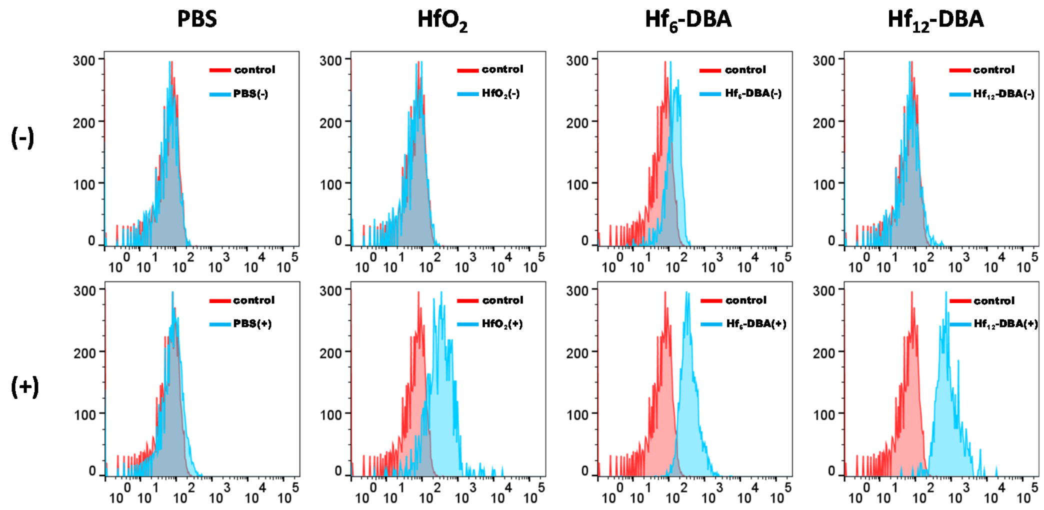

Supplementary Figure 19 DNA double strand break of CT26 was assessed after incubation with PBS control, HfO_2, Hf_6-DBA or Hf_{12}-DBA, with (+) or without (-) X-ray irradiation by flow cytometry analysis. Red histogram (control) and blue histogram show the difference of γ-H2AX level in the cells. From left to right: PBS control, HfO_2, Hf_6-DBA or Hf_{12}-DBA, respectively. One of two repetitions with similar results is shown.

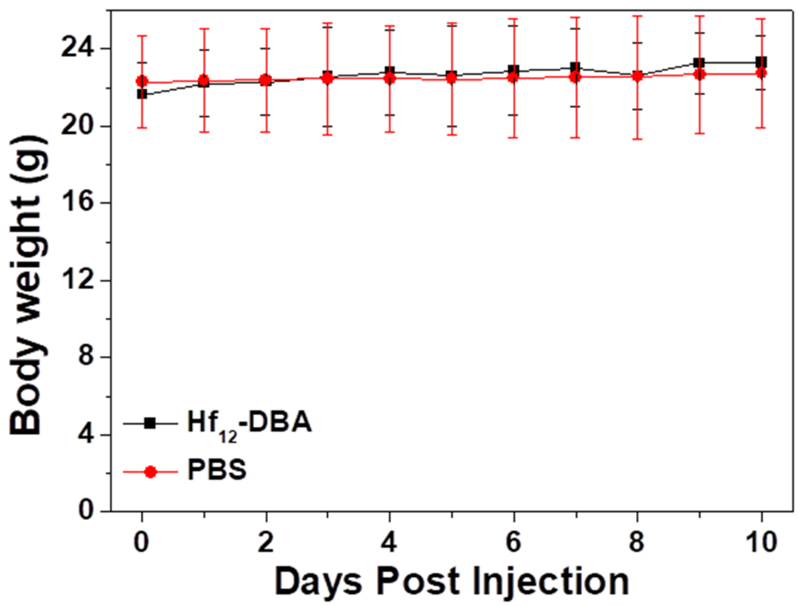

Supplementary Figure 20 Mean Body Weights of Balb/c mice treated with 10 μmol Hf_{12}-DBA per mouse or PBS. n = 3.The result was obtained without repetition. Central data points and error bars represent mean ± s.d. values, respectively.

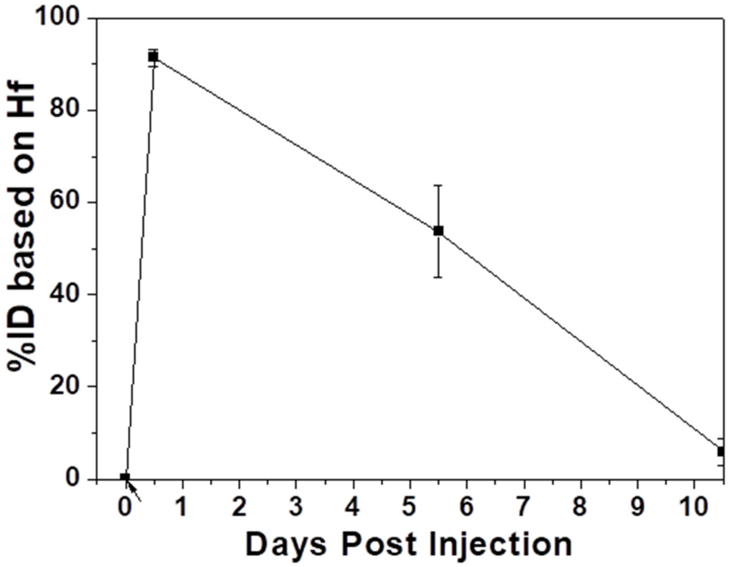

Supplementary Figure 21 Tumor retention of Hf_{12}-DBA based on Hf content after intratumoral injection to CT26 tumor-bearing mice. n = 3. The result was obtained without repetition. Central data points and error bars represent mean ± s.d. values, respectively.

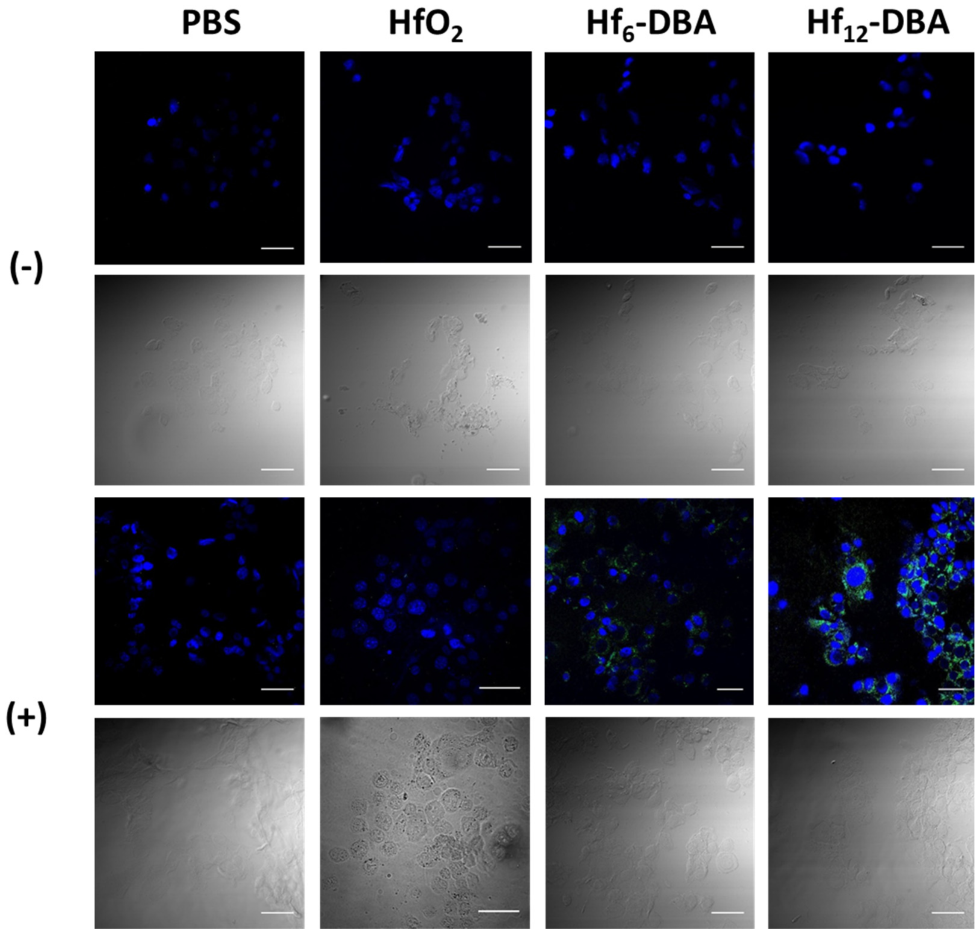

Supplementary Figure 22 Immunofluorescence microscopy of CRT expression on the cell surface of CT26 treated with PBS, Hf_6-DBA or Hf_{12}-DBA with (+) or without (-) X-ray irradiation. Blue and green fluorescence show DAPI-stained nucleus and CRT exposure on the cell surface, respectively. From left to right: PBS control, HfO_2, Hf_6-DBA or Hf_{12}-DBA, respectively. Scale bar = 20 μm. One of two repetitions with similar results is shown.

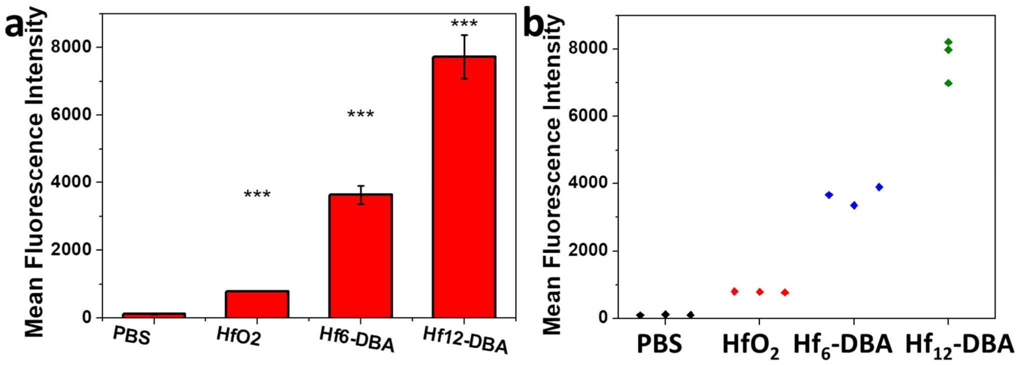

Supplementary Figure 23 Mean fluorescence intensity of CRT expression on the cell surface of CT26 treated with PBS, Hf_6-DBA or Hf_{12}-DBA with X-ray irradiation presented in (a) bar chart and (b) dot plot. From left to right: PBS control, HfO_2, Hf_6-DBA or Hf_{12}-DBA, respectively. n = 3. One of two repetitions with similar results is shown. Central data points and error bars represent mean ± s.d. values, respectively.

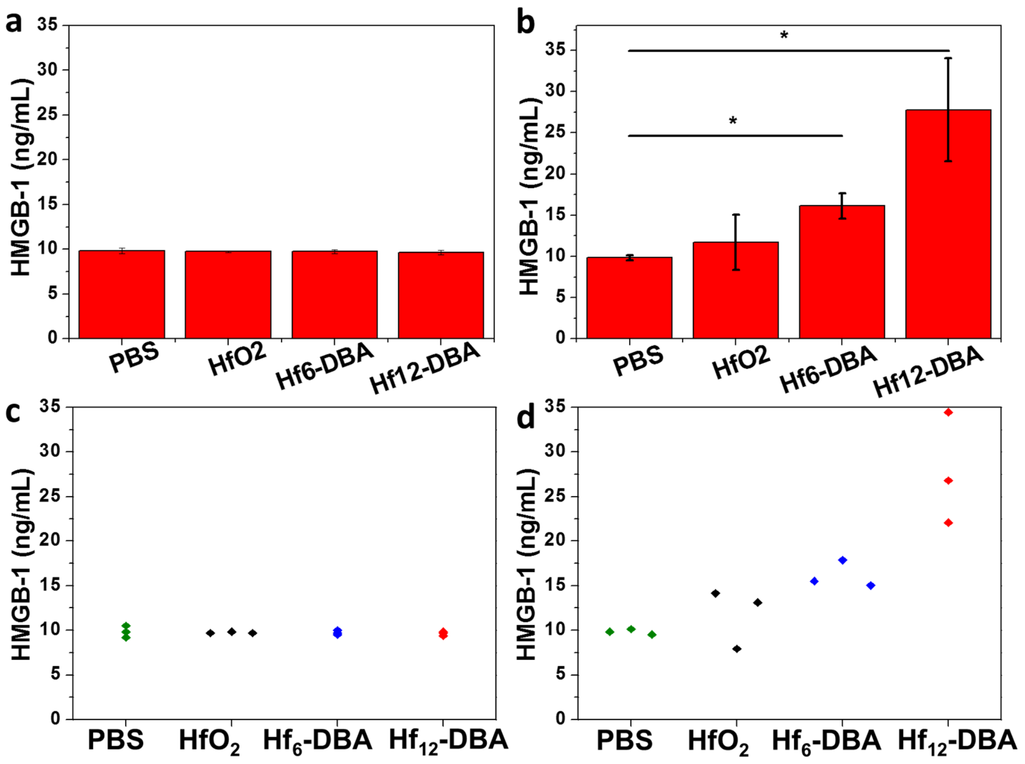

Supplementary Figure 24 HMGB1 excretion of the cells incubated with PBS, HfO_2, Hf_6-DBA, or Hf_{12}-DBA without (a, c) or with (b, d) X-ray irradiation at a dose of 4 Gy. n = 3. The result was obtained without repetition and presented in (a, b) bar chart and (c, d) dot plot. Central data points and error bars in (a) and (b) represent mean ± s.d. values, respectively.

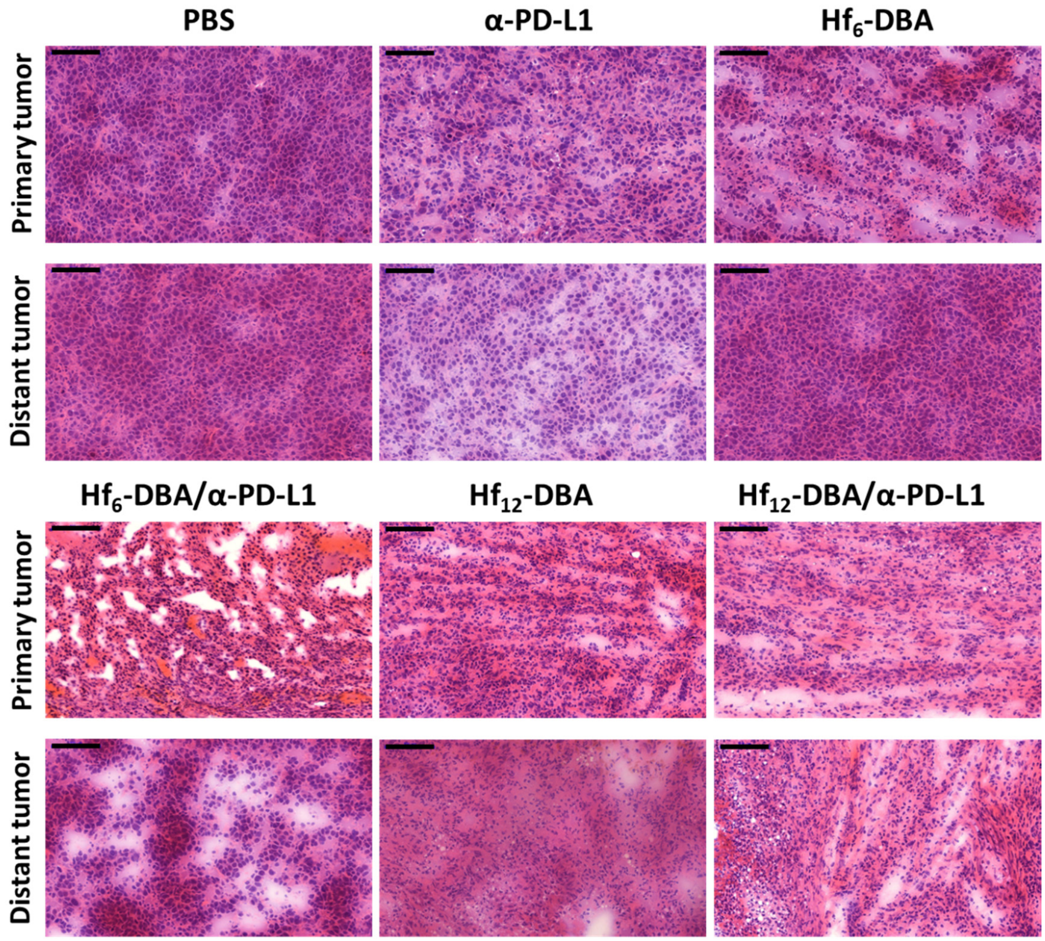

Supplementary Figure 25 Histologies of tumor slices of bilateral CT26 tumor-bearing mice receiving intratumoral injection of Hf-based nMOFs or PBS and X-ray irradiation treatment with or without antibody treatment after H&E staining. From left to right: PBS control group, α-PD-L1, Hf_6-DBA, Hf_6-DBA/α-PD-L1, Hf_{12}-DBA or Hf_{12}-DBA/α-PD-L1 treated group. Top: primary tumor; Bottom: distant tumor. Scale bar = 100 μm. The result was obtained without repetition.

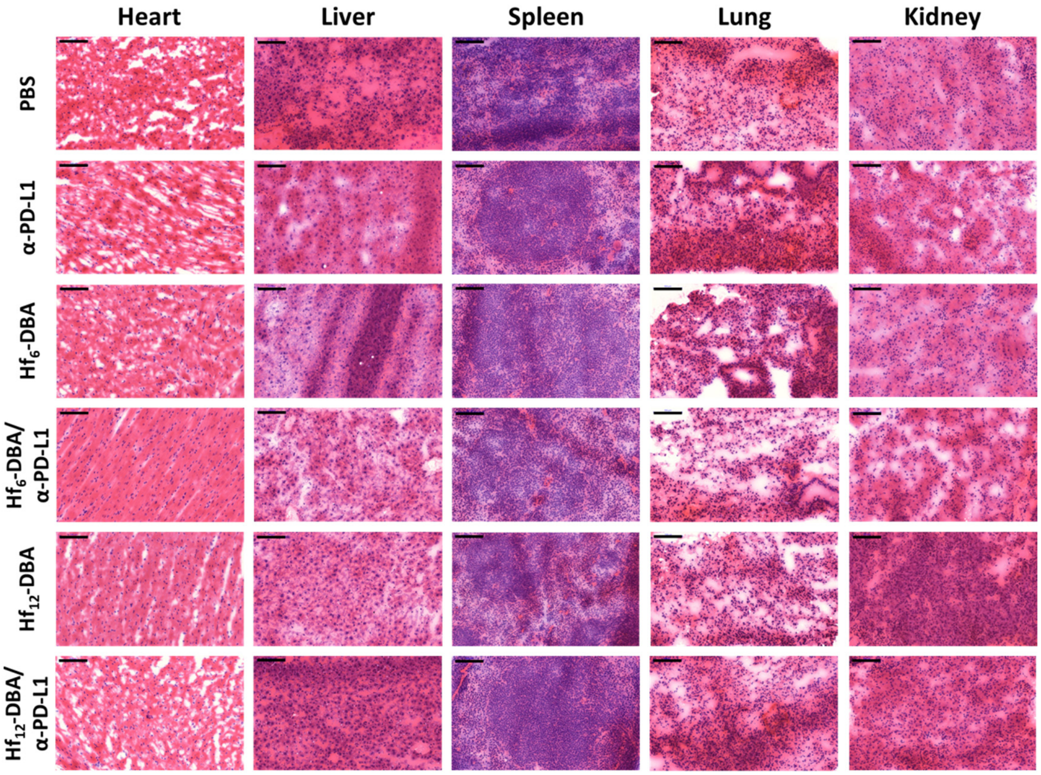

Supplementary Figure 26 Histologies of frozen sections of major organs of CT26 tumor-bearing mice receiving intratumoral injection of Hf-based nMOFs or PBS and X-ray irradiation treatment with or without antibody treatment after H&E staining. From left to right: heart, liver, spleen, lung or kidney. Scale bar = 100 μm. The result was obtained without repetition.

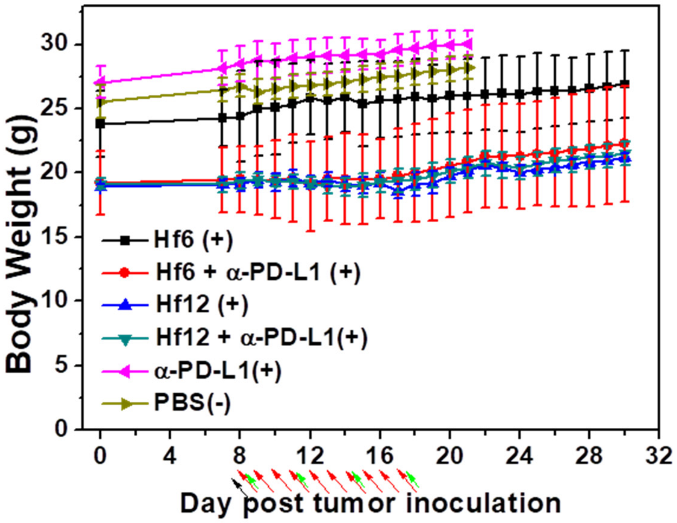

Supplementary Figure 27 Body weights of mice after X-ray irradiation treatment on bilateral CT26 models treated with PBS, Hf_6-DBA or Hf_{12}-DBA with or without anti-PD-L1 antibody. Black arrows refer to subcutaneous injection, red arrows refer to X-ray irradiation and green arrows refer to antibody injection. n = 6. The result was obtained without repetition. Central data points and error bars in (a) and (b) represent mean ± s.d. values, respectively.

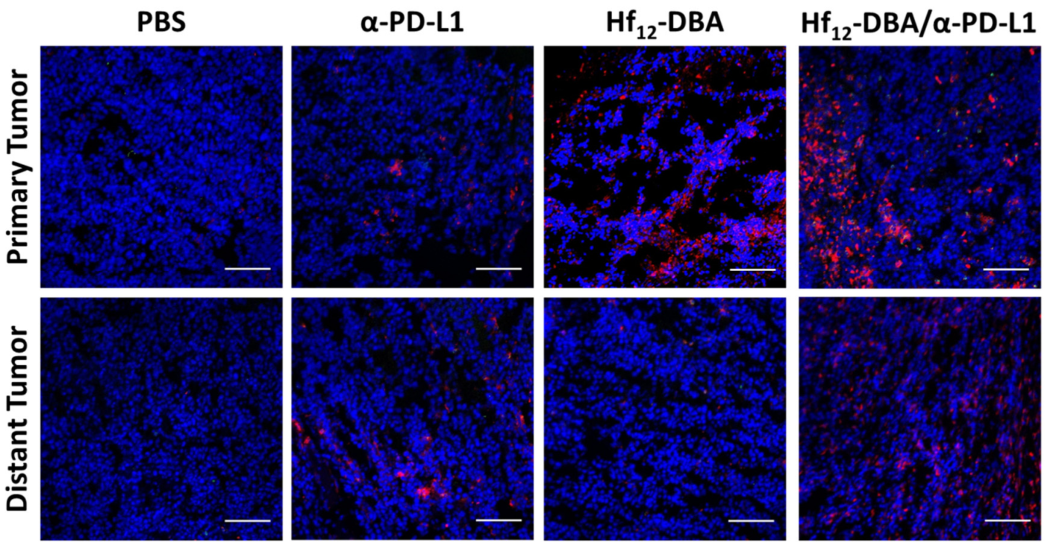

Supplementary Figure 28 T cell immunofluorescence assay. Bilateral tumor models of CT26 were established and treated with PBS or Hf_{12}-DBA with or without anti-PD-L1. 15 days after the first treatment, primary (right) and distant (left) tumors were collected, sectioned and subjected to immunofluorescence staining. Representative CLSM images of tumors after immunofluorescence staining. Red fluorescence indicates $CD8^+$ T cells. Scale bar = 100 μm. The result was obtained without repetition.

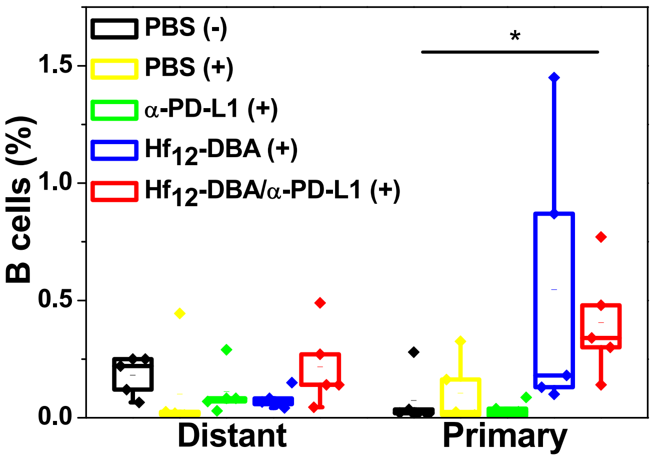

Supplementary Figure 29 Percentages of tumor-infiltrating B cells with respect to the total tumor of cells treated with PBS dark control, PBS, anti-PD-L1 antibody, Hf_{12}-DBA or Hf_{12}-DBA plus anti-PD-L1 antibody with X-ray irradiation. n=5. *P<0.05 from control by t-test. Central lines, bounds of box and whiskers represent mean values, 25% to 75% of the range of data and 1.5 fold of interquartile range away from outliers, respectively.

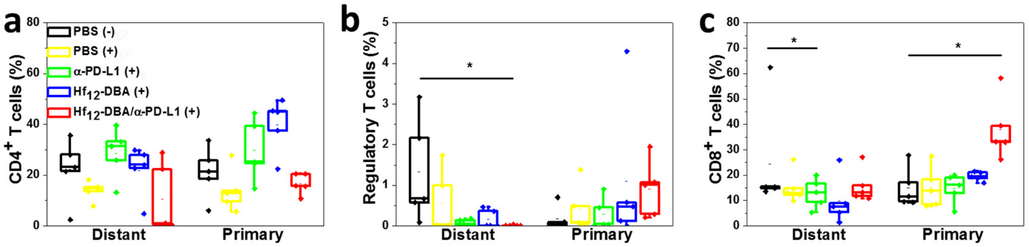

Supplementary Figure 30 Percentages of (a) CD4$^+$ T cells, (b) Regulatory T cells and (c) CD8$^+$ T cells in lymph nodes of CT26 bilateral tumor bearing mice treated as described in Figure 6 (a) and 6 (b). n=5. Central lines, bounds of box and whiskers represent mean values, 25% to 75% of the range of data and 1.5 fold of interquartile range away from outliers, respectively.

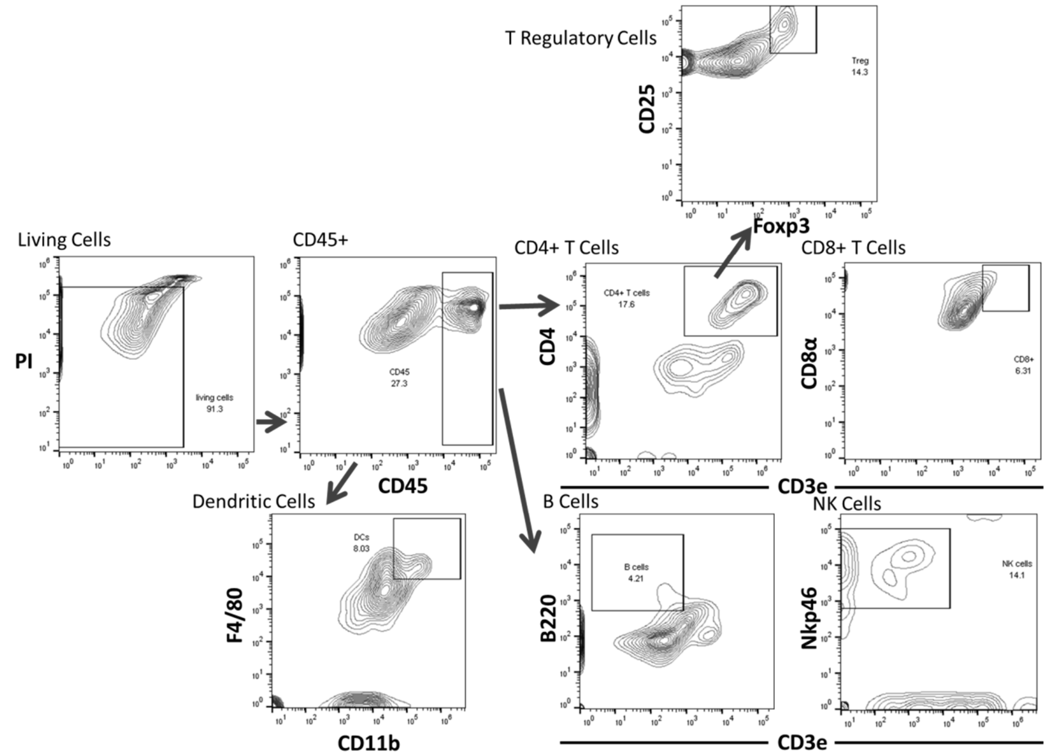

Supplementary Figure 31. Representative gating strategies for CD4$^+$ T cells, CD8$^+$ T cells, T regulatory cells, B cells, NK cells and dendritic cells.

Supplementary Table 1. Linear Fitting results of radioluminescence for Hf_6-DBAn and Hf_{12}-DBAn.

	slope	Adjusted r^2
Hf_6-DBAn	0.86±0.04	0.99
Hf_{12}-DBAn	1.36±0.05	0.992
